# Spirit of the English Periodicals, and Notices of English Medical Literature

**Published:** 1836-07-01

**Authors:** 


### Periscope;

OR,

### CIRCUMSPECTIVE REVIEW.

" Ore trahit quodcunque potest, atque addit acervo."

I.

### Spirit of the English Periodicals, and Notices of English Medical Literature.

### Modus Operandi of Medicines. By James Johnson, M.D.

*(From a Discussion in the Westminster Medical Society.)*

The experiments and observations of Dr. Addison have been now seven years before the public, and have been approved and appropriated by some of our best Toxicologists. I fully agree with Dr. A. that all violent *poisons* act through the medium of the nervous system *alone*, many of them destroying life before there is a possibility of their entering the circulation. This has been long acknowledged. I have seen the ear of a rabbit exposed to the bite of a Cobra de Capella, with a pair of scissars kept across the ear, ready to cut it off the moment the bite was inflicted; yet the animal died quickly in convulsions. But this rapid modus operandi of *poisons*, may not apply, perhaps, to the action of medicines which operate slowly, and which enter into the circulation.

It is now twenty years, since a distinguished physician, still alive, taught and published the identical doctrine of this sympathetic, or nervous agency of medicines, founded on experiments made on animals twenty years previously to that period. The following extracts are made from the second volume of Dr. Chapman's Treatise on Therapeutics.

" The theory I shall propose, of the operation of medicines, alleges, that they all act by exciting a local impression, which is extended through the medium of sympathy." *Vol.* 1, *page* 60.

Again.—" All changes in the condition of the fluids are wrought by impressions made through the medium of the solids. Not the slightest proof exists, of their undergoing any mutations from the introduction of foreign matters—much less that such is the cause of disease, or the mode in which medicines operate." *Page* 61.

3rdly.—" The process of assimilation, whether performed by the chylopoietic viscera, or by any part of the absorbent apparatus, completely decomposes all substances, however discrepant in their properties, and reduces them to a homogeneous fluid fitted for the purpose of nutrition." *Page* 63.

Lastly.—" Conformably to the theory I have adopted, whenever a medicinal substance is applied to a susceptible portion of the body, externally or internally, an action is excited, which is extended more or less, according to the diffusibility of the properties of the substance, or the degree of sympathetic connexion, which the part may maintain with the body generally. Thus a set of actions is raised, every one of which is precisely similar, provided they are confined to the same system. If the chain runs into other systems, it loses its homogeneous character, and the actions are modified by the peculiar organization of the parts in which they take place." *Page* 70.

The experiments, from which these conclusions are drawn, were conducted by Drs. Chapman and Lee, some 40

years ago, on dogs and cats, in the Pennsylvania Hospital.

These deductions appear to rest on two assumptions—neither of which is, I suspect, tenable. *First,* that no medicinal substance can be detected in the blood—and *secondly,* that the chyle is the same, or nearly so, whatever may be the nature of the food or physic taken into the stomach.

Dr. Christison, who is a disciple of Dr. Addison and Mr. Morgan, and warmly espouses their views, acknowledges that "poisons, after being swallowed, have been detected in the blood, the secretions, and the excretions." Grognier found muriate of ammonia in the serum of horses poisoned by it. Tiedemann discovered verdigris and superacetate of lead in the blood of the veins, in similar circumstances. Lebkuchner found camphor in the blood of the vena cava. Meyer detected hydrocyanate of potass, not only in the blood, but in the serous secretions of animals poisoned with that substance. Jourdan and others discovered mercury in the urine of persons who were taking it medicinally. Cantu detected iodine in the blood, sweat, urine, saliva, and milk of patients who were using it in medicinal doses. Kumer found hydrocyanic acid in the blood of animals poisoned by it. "The spirituous odour," says Dr. Christison, "of men and animals under the influence of alcohol, is a proof of the presence of that poison throughout the blood."

These are the facts adduced and acknowledged by Dr. Christison, who, nevertheless, supports the doctrine that poisons and remedies act, *solely* through the medium of the nerves. But supposing that neither poisons nor medicines could be detected in the blood, after being taken into the stomach, would that prove that neither they nor their compounds circulated with the vital fluid? Let us hear an experiment performed by Drs. Christison and Coindet. I use Dr. C.'s words. "We injected into the femoral vein of a dog eight grains and a half of oxalic acid, which caused death in half a minute. Here it was impossible that the poison could have passed off by any of the excretions; yet we could not detect even that large proportion in the blood of the iliac vein and vena cava collected immediately after death." *Toxicology, p.* 14.

We cannot, I think, draw any line of demarcation between food and physic, when taken into the circulation, as they are proved to do, whether changed or unchanged. Nor do I think that we have any experiments to prove that they do not modify the state of the circulating fluids, and influence the secerning vessels and their products, as well as the nerves of the organs through which they permeate.

We must remember that the blood contains the pabulum of all the secretions, and as the minute secreting as well as absorbent vessels are endowed with the power of selection, is it not reasonable to suppose that they will be influenced by medicinal substances, or their elements, when presented to their mouths? We rub mercurial ointment on the thighs till ptyalism is produced, and the whole of the glandular system, throughout the body, is brought into a state of great activity, with considerable changes in the various secretions and excretions. Are we to infer that all this is produced by sympathy between the nerves of the thigh (where the first impression is made), and the nerves of all the glands in the body?

Iodine is rubbed on the surface, or taken slowly into the stomach for a considerable time—and the mammæ waste. Is this from sympathy of the cutaneous or gastric nerves with those of the breasts?

Dr. A. T. Thomson detected iodine in the urine, saliva, and perspiration of patients taking that medicine in medicinal doses, and shewed some beautiful experiments in the Westminster Medical Society. The medicine is easily detected by starch and nitro-muriatic gas or acid, applied to any fluid containing even the minutest quantity of iodine. The fluid turns blue.

A very talented young chemist (Mr. Bird), stated that he had detected iodine in the blood by means of the same tests.

Drs. Addison and Chapman seem to think it unphilosophical to consider the modus operandi of medicines a compound operation, when that modus may be accounted for by a single process. Why should the blood and the blood-vessels, say they, have any thing to do in the cure of syphilis by mercury, when the whole process can be explained by the nerves? Can mercurial ointment rubbed on the legs or thighs, cure a chancre on the penis, simply by sympathy between the nerves of the two parts? It is curious that this sympathy very seldom produces any curative effect till the gums become sore, and the breath exhibits a strong mercurial fetor.

The poison of syphilis *ought* to be a stumbling-block to the simple and *solely* nervous agency of poisons in all cases. Does the application of the syphilitic virus to the glans penis produce, not only the chancre, but the subsequent affections of the bones and other parts, *solely* by sympathy between the nerves of the glans penis and all these different structures? Is it not more probable that the poison passes into the system by means of absorption, tainting the whole mass of blood, and diseasing various tissues for which the virus had an affinity or predilection?

The liver is torpid and the stools are like clay. Mercury is taken for five or six days, till the mouth is touched, when the bile flows freely, and the stools become yellow. Yet all this is supposed to be from sympathy between the gastric and hepatic nerves, *alone*.

Drs. Addison and Chapman will, no doubt, explain these phenomena by supposing that, when mercury is taken, it is the nerves of the liver or other glands that set the secretions in activity. Allowing this to be the case, as I do, it only gives the nerves the *initiatory* in the process; the blood and the secerning vessels being essentially involved and employed in that same process. I do not deny the agency of the nerves in every curative as well as destructive operation; but I deny their exclusive agency. The nerves cannot secrete, without the vessels, nor can the vessels without the nerves. It is their joint agency for which I contend—it is their insulated or independent actions, in the MODUS OPERANDI of medicines, that I repudiate.

Where do we see any great process in the animal economy, whether conservative or destructive, carried out by the nervous system, exclusive of the vascular—or by the vascular independent of the nervous? Is digestion, assimilation, sanguification, secretion, or absorption, performed by one of these systems alone, or in any case? And is the MODUS OPERANDI of medicines, (which are only auxiliaries or correctives of some of these processes) to be made an exception, and an anomaly in the operations of the living machine?

Is the chemistry of the animal fluids, to be placed entirely to the account of the nervous system? We have turbid urine, with deposition of uric acid. A few drachms of soda change the state of renal secretion, and render it clear. Is this from sympathy, and from sympathy *alone*, of the nerves of the stomach with those of the kidney?

A man has epilepsy, and he takes nitrate of silver for several months. The disease may or may not be cured; but the skin becomes permanently blue. Is the MODUS OPERANDI of argentum nitratum to be solely attributed to sympathy between the nerves of the stomach and the skin?

I am ready to grant, what, indeed, no physiologist or pathologist will deny, that the primary impressions of all kinds of food, and all kinds of physic, are made on the nervous system—and that violent poisons will destroy life through this system alone, as a blow on the stomach will kill instantaneously, before the vascular system can be called into play. But this I maintain, that, when medicines are slowly introduced into the body, they excite a series of actions in the nervous and vascular apparatus, CONJOINTLY and not SEPARATELY,—and that it is through this conjoint agency, (whichever may take precedency) that all remedial or deleterious effects are produced.

There is, after all, but little real difference between Dr. Addison and Dr. Johnson. The chief difference is this: The *former* thinks the entrance of *medicines*, as well as poisons, into the circulation, unnecessary : — The *latter* thinks that medicines, and poisons in medicinal doses, do enter the circulation before their full remedial agency can be effected—always excepting, of course, those medicines which act, and are intended to act, *locally*, as purgatives, &c.

### Proposed Modus Operandi of Medicines.

I submit that, in cases where medicines are exhibited *in medicinal* doses, viz : for the remedy of disease, and not for the destruction of life, the MODUS AGENDI consists of three distinct and consecutive processes, as far as the evidence of our senses is concerned.

*First.* The physiological action of the medicine itself on the nervous system of the part or parts to which it is applied.

*Secondly.* The physiological action or re-action of the nerves on the vascular, glandular, or fibrous tissues of the same part or parts.

*Thirdly.* The physiological action induced in the vascular, glandular, and fibrous part or parts, by the two preceding processes—in other words, the remedial *effects* or *products* of the medicine employed.

Now, of the *Modus Operandi* of the medicine on the nerves—and of the nerves on the vessels, glands, and muscles, we are entirely ignorant—and we only take them for granted ; and of the third process—the physiological action of vessels, glands, &c. we only become cognizant, by the effects or products of this action. To illustrate this reasoning let us take an example.

Calomel is introduced, in successive doses, into the stomach. We know not its physiological mode of action on the nerves of that organ, or of any other organ to which it may be carried. Neither do we know the modus operandi of the ganglionic nerves on the vessels, glands, or other tissues of the parts which they supply. But the third process takes place—the physiological action of these last structures, and then we have the visible, tangible, demonstrable results—ptyalism—increased action of all the glands—alteration in the various secretions and excretions.

In these three consecutive processes, then, this *tria juncta in uno*—we have the modus operandi of medicines. The medicinal agency is the first—the nervous agency is the second—the vascular agency is the third.—Any one of the agents being absent (I mean in therapeutics) the others cannot act. In the exhibition of medicines, all the three processes must be called into play—and the result is, the MODUS OPERANDI, in a practical point of view.*

The only question that remains, and in this I differ from the exclusive sympathetic doctrine, is this :—Is the nervous system of the various parts remote from the seat of primary impression—(the stomach, for instance, when mercury is exhibited)—excited into action by purely nervous sympathy with that primary part, or by the presence of the mercury, however decomposed or modified, carried to these parts by the circulation ?—This is the problem to be solved—and my own opinion is, that the probability is in favour of the latter

---

* It is quite useless, in the present state of our knowledge, to attempt an explanation of the *mode or manner*, in which nature operates, whether in respect to poisons or remedies. The *effects* of these agents are all that we can ascertain. The effects, then, of poisons, in large and in small doses, are different. Small doses of arsenic will cure diseases, and produce a train of phenomena totally different from those produced by large or poisonous doses. How, then, can we say that the modus operandi must be the same in both cases? Thirty grains of calomel will purge—and do nothing else, in a great majority of cases. The same given in grain doses, at intervals of eight hours, will salivate and affect the whole system. Is the *modus operandi* the same in both instances? Is not the mercury taken into the system, in the *latter* case, and does it not act, through the medium of the blood, on the whole machine? In the *former* case, does it not act on the intestinal canal without absorption?

supposition—namely, that the mercury (and I only take this as a familiar example) permeates throughout the whole system, and acts on each individual organ and part (through the nerves and vessels), in the same way as it did on the part to which it was first applied.

Lastly.—If the physiological action of medicines were propagated from the stomach to other organs and parts, by nervous sympathy *alone*, that action ought to be first and most strongly manifested in those organs and parts with which the stomach most readily sympathizes. But this is so far from being the case, that we can never predicate the direction which any particular medicine may take, till it is determined by experience. Who could have prognosticated that antimony would vomit, colocynth purge, mercury salivate, iodine emaciate, digitalis depress, and bark invigorate, by any investigation of the physical or chemical characters of these and a hundred other officinal substances? I contend, then, that the laws by which medicinal agents are directed to particular organs and parts of the living body, are, as yet, unknown—and that the MODUS OPERANDI, by nervous sympathy *alone*, does not satisfactorily account for the phenomena which we observe in the practice of physic.

P. S. We regret that we are unable to give Dr. Addison's able speech on this subject. It was not written, and it was delivered with the usual eloquence and rapidity of the talented Chairman of the Westminster Medical Society.—EDS.

---

QUACKERY; ITS DANGER, IRRATIONALITY, &c.

This little brochure, of sixteen or eighteen pages, is remarkably well written; but, as addressed to the community at large, will have about as much effect against quackery, as the best sermons have against sin—viz: no effect at all! Our legislators have always been chary of abridging John Bull's liberties, even where these extended to self-destruction. They tax his purse freely—but allow him to be poisoned by every unprincipled Charlatan that comes in his way. There can be no cure for the evil, except in the restrictive laws adopted by Continental Governments; and it is very doubtful whether our collective wisdom will ever consent to such a serious inroad on our freedom, as the suppression of quackery would effect. There is no harm, however, in the getting-up of petitions to Parliament. The following form, appended to the pamphlet, is, we think, as good a one as can be devised.

"That the object of medical science is the establishment of fixed principles, to regulate the application of remedies in the cure and prevention of disease; and it is contrary to all reason and analogy to suppose that the necessary knowledge can either be acquired or properly applied without minute and accurate acquaintance with the healthy and morbid conditions of the human frame.

That in order to furnish the means for attaining this desirable end, and for securing the best possible qualifications in those entrusted with the important responsibility of ministering to the public health, universities and societies have been formed; an arduous and expensive course of education enjoined; and penalties attached to the infringement of regulations, which enlightened experience and legislative enactment have found to be essential, though hitherto insufficient safeguards, against the misdirected and injurious efforts of the ignorant or unprincipled Quack.

That while the utility of the medical profession is thus publicly recognised, and much laudable anxiety shown for securing the attainments and respectability of its members, its privileges are glaringly and unjustly infringed by the success and pretensions of unqualified pretenders, whose assertions, while they are repudiated both by reason and experience, exert a powerful and deadly influence over the health of a too easily deluded population.

That the absence of any legal means for the suppression of Quacks and Quack Medicines, and the positive encouragement afforded them by the system of stamps and advertisements, either implies the *inutility* of qualified medical men, or involves a flagrant violation of their rights, by depriving them of that protection and consideration which a just regard for the public health imperatively demands, and to which, as a formally authorised and privileged body, they are legally entitled.

That the public having no means of discriminating between the spurious and properly qualified medical practitioner, it urgently behoves Government itself to provide, as far as possible, for the health of the community, by extending efficient protection to the privileges, while it enforces evidence of the acquirements and capabilities of those who are entrusted with so important and difficult a charge.

It is therefore humbly proposed, that all Patent, Quack, and Secret Medicines be abolished.

That no medicine be advertised, the real composition of which is not appended, and its efficacy properly attested.

That no individual (druggist or others), who has not complied with existing regulations, be allowed to exercise the healing art; and that all establishments nominally devoted to the same purpose, but conducted by persons not properly qualified, be declared illegal.

That all established medical bodies shall have the power of expelling from their halls, and expunging from the list of their members, all those who have recourse to unprofessional modes of gaining notoriety. And your petitioners will ever pray, &c."

---

### Carbonate of Soda in large Doses. By Mr. Neville.

Mr. Neville has recently published a small, but very well written volume on mental derangement. But, as the greater part of it is eclectic, or, in other words, compiled from the best authorities, it is unsuitable for a review—being itself, indeed, an analysis. There are several original remarks, however, and observations scattered through the volume, and the following passage contains some curious matters which we deem worthy of selection. It is on the exhibition of soda, as a corrector or preventive of indigestion and its multiplied horrors.

"We selected two healthy young monkeys, nearly of an age. We preferred them to any other, in this instance, for experiments, on account of their similitude to man in their omnivorous capacities, as also in their passions. These creatures we had placed precisely in the same circumstances, and treated as nearly as might be in the manner in which we are accustomed to treat ourselves in society; that is to say, we had them placed in a comfortable warm room, gave them little opportunity for exercise, dieted them, according to the fashion of the times, with an abundance of the staple articles which constitute the food of an Englishman: they had tea, bread and butter, with some animal food, for breakfast; meat, vegetables, fruit pies, raw fruit, beer, and wine, for dinner; and tea and supper as these meals are taken among the middling classes of society. In short, they were treated every way alike; save that to the one, and not to the other, a drachm and a half of the carbonate of soda, dissolved in water, was regularly administered in three doses during the course of every day. At the end of six months the two animals presented a very different exterior, and were evidently in dissimilar states of health. The one to whom the soda had been given was plump and lively: the other was thin, spiritless, and obviously diseased. We had this creature killed; when, upon examining its body, we found the muscles to be shrunk and pale, the stomach and alimentary canal congested, the mucous coat of the former especially thickened, the right lobe of the liver indurated, and the mesenteric glands much enlarged.

For fear the above result might have been occasioned by circumstances with which we were not acquainted, or over which we had no control, the same experiment was repeated, with precisely similar effects, on two other animals of the same species; and also a third time, upon two strong whelps of the Newfoundland breed of dogs, without any material difference being observable. The carbonate of soda appeared to prevent the accession of disordered function in each instance:—the animals that were pampered and over-fed, without having it administered to them, became sickly, and pined; those who took the salt three times a day throve, and enjoyed to all outward appearance the best state of health; nor, upon dissection, could any trace of disease be found in their bodies, which was always very apparent in those of the others."*

The author relates some instances of the beneficial effects of soda in children. The following is an example.

"Twin boys, very healthy children, were placed, as nearly as might be, in similar circumstances, with two different and excellent dry nurses. One child was observed to thrive amazingly; but the other looked puny, and was evidently in indifferent health. Upon expressing our particular satisfaction at the appearance of the robust infant one day, the nurse, with a very complacent look, begged to assure us that 'it all came of her bottle.' On analysis of this medicine; we found it to contain a mixture of liquor potassæ, infus. rhei, aqua anethi, and simple syrup. This mixture she was in the habit of giving to her little charge in the dose of a tea-spoonful night and morning, and more frequently still when he did not seem quite himself; though the original instructions prescribed only a few drops."

A gentleman, aged 40 years, had suffered so long and so severely from indigestion, that his life became a burthen to him. He was persuaded by our author to take "two drachms of carbonate of soda, twice or thrice a day, in a bitter infusion." Though startled at the magnitude of the dose, he made the trial. Five months afterwards, Mr. Neville learnt from this gentleman that, on the very first day of the trial, he felt himself a new man. At the date of the letter, he was eating and drinking, *ad libitum*, having recovered his strength, and grown hale and stout.

"A lady of distinction, whom we visited for some time, along with her ordinary medical attendant, a practitioner of eminence, suffered from indigestion nearly or altogether in as great a degree as the gentleman whose case we have mentioned above. All dietetic means, combined with the exhibition of alteratives, aperients, small doses of the alkalies, &c. had also proved utterly unavailing in his instance. At our earnest request, the patient was prescribed an ounce of carbonate of soda, divided into five equal portions, in the course of the four and twenty hours. Under this medicine alone she rapidly and completely recovered, regaining all her former spirits, and once more presenting the plumpness and bloom of perfect health."

There is no doubt that acidities in the stomach and duodenum are productive of singular irritability in the mind and the whole of the nervous system. But we much doubt whether the carbonates of soda and potash can be taken in the above doses with impunity. We have known several instances where much smaller doses taken regularly have appeared to render the mucous membrane of the bowels more instead of less irritable. The constant use of it appears to us to clear away the mucus which is the natural defence of these parts against foreign matters that must pass along their surfaces.

* Neville on Insanity, p. 174-6.

---

### The Philosophy of Decapitation.*

The sublime soliloquies and meditations of Hamlet and Macbeth are likely to be renewed in the medical world. Macbeth, with sorrow and alarm, remarks:—

> "Time was,

> That when the brains were out, the man would [die;

> But now!"

* Dr. Rigby—Med. Gazette, No. 435, April 1836.

Hamlet philosophises in a still more exalted strain.

[come,
"Yet in that sleep of death, what dreams may
When we have shuffled off this mortal coil,
Must give us pause."

The great physiological question that now agitates the breasts of phlegmatic German and imaginative Frank, is the state of sensibility and moral feeling after decapitation by hatchet, sword, or guillotine! It is well known that "Charlotte Corday's face blushed after decapitation, when the executioner struck it a blow on the cheek," though some sceptics have affected to doubt the truth of this authentic fact, as they doubt, indeed, even the great truths of Holy Writ. The ingenious author of this paper (which was read at the College of Physicians) quotes largely from Dr. Nasse of Bonn, and he thinks it is not difficult to explain, "both on physiological and psychological principles, that the head must die after decapitation." But he says that it is by no means so easy to explain how and why the head dies, when thus suddenly divorced from the body. When we consider that cessation of the heart's action, even for a few minutes, destroys the life of the individual, head, body and members, we can be at very little loss to conceive how and why the head should die, when it receives no more blood from the heart—and loses a good deal of what circulated in its vessels. But passing over this how and wherefore, we come to some staggering proofs that the decapitated head (which is rather an Hibernicism) preserves for some time a kind of independent existence, contemplating, with various emotions, the assembled spectators—expressing surprize at certain sounds and words—turning the eyes after particular objects—and, in short, all but entering into conversation with the priest and the executioner! Various proofs of this are authentically recorded. The lips of Mary Queen of Scots continued praying (not audibly, but visibly) for a quarter of an hour after decapitation. "Wendt relates that, having put his mouth to the criminal's ear, and called him by name, the eyes (of the decapitated) turned to the side from which the voice came." The authorities of Fontenelle, Mogon, Guillotin, Nauche, and Aldini, are quoted to prove the same *fact*.

"As the word 'MURDER' was called into the ear of a criminal, who was executed for this crime at Coblentz, the half-closed eyes opened wide, and he stared, with an expression of astonishment, at those who stood before him."*

If honest DAN, the member for all Ireland, were to hear such a statement, his observation would, doubtless, be—"this beats Bannagher, and Bannagher beats the ———."

Now we do not accuse the learned author of the paper of believing one word of these and various other well-authenticated facts:—on the contrary, Dr. Rigby is evidently a sceptic—and so, we believe, is every one within the confines of the British Isles, who has a brain the size of a pineal gland. We believe that, since STEVENS' celebrated "Lecture on Heads," there has not been a more capital hit than this discourse on decapitation, especially when the place of its delivery is taken into account. The Temple of Esculapius, in Pall-mall East, is becoming quite a tragic theatre—a Golgotha—an Abattoir—where the deaths of kings, and the decapitation of criminals, are enacted with classic eloquence, and pathological philosophy.

---

ON RUPTURE OF THE HEART. By Dr. HALLOWELL, OF PHILADELPHIA.†

Dr. Hallowell, who appears to be a well informed and judicious young physician of America, has published a very creditable paper on Rupture of the Heart, in our Trans-Atlantic contemporary. The paper is too long for insertion in our columns, but we will present a sketch of the leading facts and observations contained in it. The compara-

* Med. Gazette.

† Amer. Jour. of the Med. Sciences.

tive rarity of the disease, and the imperfect accounts which we possess of it, induce us to extend the present notice beyond what we might otherwise do. Dr. Hallowell observes:—

"Spontaneous rupture of the heart is of rare occurrence. Neither CORVISART, LAENNEC, BERTIN, nor SENAC, whose experience in cardiac diseases was very great, met with a single case of it. The number of well-attested observations amounts perhaps to sixty. I have collected thirty-four of them in various publications, with but little research. In the history of these cases, which is often very imperfectly detailed, it is stated that the patients had been affected for a greater or less length of time, with palpitations, and had experienced frequent attacks of lipothymia, or complained of pain beneath the sternum and tightness and weight across the chest, &c. these symptoms being no doubt dependent upon one or more of the morbid conditions to be hereafter noticed."

Death usually takes place suddenly; but instances have been recorded in which hours, nay, we are told, even years, have elapsed, between the cardiac rupture and death.

The accident usually occurs in persons in advanced life. Of the thirty-four cases collected by Dr. Hallowell, the age is precisely stated in twenty-three only. Of these, nine were between seventy and eighty, six between sixty and seventy, five between fifty and sixty, two between forty and fifty, and one between twenty and thirty, (from dilatation.) Sixteen of the thirty-four individuals were males, and eighteen were females.

The rupture occurs, for the most part, in the left ventricle, in its anterior wall near its middle. In the above-mentioned cases, the place of rupture is stated in thirty-one. Of these there were three ruptures of the right auricle, none of the left, two of the right ventricle, and the remaining twenty-six of the left ventricle.

The rupture varies in size from an almost imperceptible aperture, to a slit an inch or more in length. It may be single, or there may be several.

The tissue of the heart, surrounding the place of rupture, will be found in one of the following conditions.

1. In a perfectly healthy state.
2. In a state of ulceration.
3. Hypertrophied with or without ramollissement or softening.
4. Softened to a greater or less extent.
5. Dilated and thinned.
6. To have become the seat of a partial dilatation.
7. To have undergone a fatty degeneration.

On each of the preceding states Dr. Hallowell offers some remarks.

1. Dr. Hallowell has never met with a perfectly satisfactory example of rupture of the heart independently of any alteration of its tissue.

2. Numerous cases of rupture dependent on ulceration of the heart are upon record. Omitting many mentioned by our author, we may notice two instances in our Royal Family. George II. died of rupture of the heart in the act of defæcation; and the Duchess of Brunswick died of the same malady. On examination, there was found an ulceration through the walls of the right ventricle, which had commenced externally and gradually penetrated inwards.

The ulceration may commence on the external or the internal surface of the heart.

3. "The next form is that of *hypertrophy, with or without ramollissement.* When the latter of these states exists, it is often found to be connected with ossification and contraction of the ventriculo aortic orifice. The mitral valves being closed to prevent regurgitation, it is easy to conceive, that the blood, finding no egress, is powerfully acted upon by the ventricle which, stimulated to violent contraction by the double impulse of powerful mental excitement, and the reaction of the mass of fluid within its cavity, may give way, and the blood be effused within the pericardium. In one of the cases recorded, it is also stated that the arteria innominata at its origin was so diminished in its diameter by atheromatous depositions between its coats, as to leave a passage not more that one-eighth of an inch in diameter, and the left subclavian artery was in like manner diseased, and diminished one-half its calibre. Softening, associated with hypertrophy, may be either general or partial,—it may be confined to the muscular structure immediately around the rupture, or it may exist in patches. The substance of the heart in the place of rupture, is sometimes reduced into a soft yellowish pulp, without any trace of muscular fibres."

4. The heart may be simply softened to a greater or a less extent—an alteration the precise cause of which would still seem to be uncertain. In many instances it appears to be decidedly the result of inflammation; in others, it may be, as Laennec supposed, an alteration sui generis.

5. The heart may be dilated, with thinned parietes. Dr. Hallowell cites the particulars of several cases of rupture of the heart in this condition. In two, the right auricle gave way; in one, the right ventricle.

6. The rupture may result from partial dilatation, or aneurysm. Talma, laboured under this disease. M. Breschet, from an examination of ten cases of this disease, has arrived at the following conclusions.

1. That the heart may be affected with aneurysm of a different nature from that ordinarily described.

2. That this disease ordinarily affects the left ventricle.

3. That it exists most commonly at the apex of the heart.

4. That this aneurysm, from the mode of its formation, the state of its parietes, and the contents of its cavity, closely resembles false consecutive aneurysm in the sense given by SCARPA to the term aneurysm.

Passing over the detail of some of Breschet's cases, as of others, we may notice one communicated by Dr. Hallowell himself. He saw it in the Salpêtrière, at Paris.

*Case.* An old woman, æt. 82, was admitted into the hospital in November, 1832, with paralysis, dyspnœa, cough, and bloody expectoration. She had had fits of palpitation. Nothing particular was observed in the sounds of the heart. The pulse was rather feeble, and slightly accelerated. She died eight days after her admission.

*Dissection.* Passing over other appearances, we will limit ourselves to the condition of the heart. The pericardium contained a small quantity of transparent serosity. The heart was of the ordinary size, but singular in appearance, its point being very saillant, projecting anteriorly, and covered with a pretty large quantity of fat. The greater part of the fat was removed with care, which reduced the sac to a lamina of very slight thickness. The left ventricle was then opened transversely, and a second longitudinal incision made as far as the orifice of the tumor, which was about two and a half lines in diameter—a female catheter being introduced with great facility. It resembled the neck of a herniary sac; the tumor was of a roundish form, slightly conical, the point projecting forwards. Its transverse diameter measured about an inch—its longitudinal fifteen lines. Having continued the perpendicular incision to the summit of the sac, the fleshy columns of the ventricle were seen to advance within its interior, becoming much thinner. The muscular structure of the heart extended as far as the summit, being there but a line, or less, in thickness. It might almost be said that the inner surface was immediately in contact with the pericardium. Its cavity contained a quantity of liquid blood, surrounding a blackish coagulum of the size of a filbert. This coagulum did not adhere to the sac, and was not formed of concentric layers, as in aneurysm of the arteries. The left ventricle was slightly hypertrophied, its cavity appearing to be of the natural size, (excentric hypertrophy). The right ventricle seemed slightly dilated, and there was a considerable ossification about the left auriculo-ventricular orifice. Several smaller ones were observed at the ventriculo-aortic orifice, the largest in the middle sigmoid valvule. The orifice, however, was not sensibly contracted. The coronary arteries were dilated—a female catheter was introduced without difficulty.

Breschet states that these aneurysms are usually found at the apex of the heart; but in seven well-authenticated cases, the tumor was at the side or base of the ventricle.

7. Rupture from fatty degeneration of the heart is the last variety. The structure of the heart is more or less converted into a substance, possessing the physical and chemical properties of fat. Dr. Hallowell relates a case of this rare affection which came under his own observation in Paris.

*Case.* An old woman, æt. 76, was found dead in the privy of the Salpêtrière. She had supped with a good appetite on the evening previous to her death.

*Dissection.* There were ossifications of the falx cerebri, and in the inferior and posterior surface of the medulla spinalis.

"On opening the thorax, the pericardium was seen greatly distended, and presenting a black appearance. An incision being made into it, the heart was displayed, surrounded by an enormous clot of black blood. This was removed in one piece, and appeared to weigh about ℥xij. Before removing the heart from the chest, we examined, with care, the spot at which the rupture took place. There were two small linear openings, three or four lines in length, separated from each other by an interval of three lines, situated on the anterior face of the left ventricle, one-fourth of its length from the apex. An attempt was made by M. Cruveilhier to penetrate within the cavity of the ventricle through the opening, but without success. A longitudinal incision was now made through its parietes, and the cause of the obstruction at once made evident in their narrow and sinuous course, uniting near the centre, and communicating with the cavity of the ventricle by a single opening. The thickness of the wall of the ventricle was but slightly diminished; around, and a little above the rupture, in the part corresponding to the external border of the ventricle, the tissue of the heart seemed less red than in its natural state, and especially than in the interior of the septum. There was at the same time a peculiar marbled appearance, exhibiting yellow streaks, not of a roundish form, whose direction was parallel with that of the fleshy fibres of the heart. These yellow streaks were not much softer than that part of the heart surrounding them, but the substance of the heart, when cut into thin slices, exhibited a friability, comparable to that of the liver. This condition of the ventricle appeared to be a fatty degeneration. The right ventricle was covered with a considerable quantity of fat; it presented in other respects nothing worthy of note."

The muscles of the back were partially converted into fat; so were those of the legs.

The preceding sketch of Dr. Hallowell's paper may not be uninteresting or uninstructive to those who take an interest in the pathology of the heart.

---

Occlusion of Cystic Duct—Lining Membrane of Gall-Bladder changed into a Pus-secreting Surface—Gall-Bladder enormously enlarged, and filled with Pus and Gall-Stones. By James Johnson, M.D.

Mr. Tozer, a chemist in Piccadilly, had consulted me occasionally for two years or more, always complaining of deep-seated pain in the region of the liver, besides a number of dyspeptic symptoms, as heart-burn, uneasy and protracted digestion, irritable bowels, turbid urine, and unhealthy motions. He had had some attacks of gall-stones; but I never saw him in any of these attacks. The eye, and even the skin, were sometimes slightly tinged yellow. I made many and careful examinations of the seat of pain, but never could discover anything that could at all explain the nature of the complaint. Besides the usual remedies for indigestion, leeches, blisters, and even issues were employed, but with little or no relief. I became, in fact, tired out with listening to the host of miseries which my patient described, and at last thought that he was hypochondriacal and exaggerated all his dolorous feelings. On the 20th of March, 1836, however, Mr. Tozer was seized with such violent and excruciating pain, in the usual place, accompanied by fever, vomiting, and rigors, that no doubt remained in my mind, as to the presence of inflammation in some structure or other. He had, in the course of that and the next day, some sixty or seventy leeches applied besides blisters, and antiphlogistic measures of every kind. Very little relief was obtained. The shiverings recurred occasionally, and there was generally a high range of evening and nocturnal fever, with morning perspirations. A few days after this new train of symptoms there occurred such a violent diarrhœa, from some active aperient medicine, that we thought he would have died. Some discharges of blood, also took place from the bowels. The original pain continued, and no examination could detect its seat or nature. Dr. Bright and Mr. H. Johnson attended this unfortunate patient with me, and both of them came to the conclusion that abscess had taken place in the liver. I confessed all along that I was totally unable to form any clear opinion of the pathological state of the case. This unfortunate sufferer lingered, under fever, diarrhœa, and intestinal hæmorrhage, till the 14th April, when death put an end to his sufferings.

The body was examined by Mr. Johnson, in presence of Dr. Bright and myself. The emaciation was considerable; but there was very little yellowness of skin. On opening the abdomen, a globular tumor was seen projecting about half an inch beyond the thin edge of the liver, to which it was intimately adherent. When touched it fluctuated, and evidently contained a fluid. When opened a pint of thick purulent matter issued, mixed with a large quanty of biliary calculi, some quite soft, and others of firmer consistence. The parietes of the tumor or sac, were then carefully traced, and they were found to be intimately adherent, not only to the liver above, but to all surrounding parts, by ancient adhesive inflammation. They adhered to the duodenum, pancreas, and even the kidney of that side. It was clearly the gall-bladder itself, thickened, diseased, and, in some places, ulcerated. Its duct was obliterated all but about an inch—this cul-de-sac being next to the ductus hepaticus and communis. The internal surface of the gall-bladder had become a secreting surface, probably from the irritation of the calculi, and the ductus cysticus being obliterated, the secretion and distention went on till the gall-bladder had attained this enormous size.

Thus it might, in some sense, be said to have been a suppuration in the liver; but still, it was not an abscess, according to what we mean by that word generally. It was, in short, a case of biliary calculi, followed by occlusion of the ductus cysticus—purulent secretion—great enlargement of capacity. The ductus hepaticus and communis were not stopped; but the remarkable enlargement of all the branches of the hepatic ducts, even to the pori biliarii, shewed that free egress of the bile from the liver had not always obtained. The structure of the liver itself was not altered, though the parietes of the gall-bladder appeared, in some places, eroded through, where they were adherent to that of the hepatic structure.

Notwithstanding the long-continued diarrhœa, and repeated discharges of blood from the bowels, there was no ulceration or other disease in those parts.

---

### Paralysis of the Tongue and Muscles of Deglutition, without Apoplexy or any Diminution of the Sensorial Functions. By James Johnson, M.D.

A lady (Mrs. W.) sister of the celebrated Mrs. Siddons, aged 76 years, had been subject to pain in the back of the head, for 20 years or more. She had also had a distressing and buzzing sound in the ears, especially in the left ear—but, in other respects, she enjoyed general good health. About two years before the date of my present visit

(Monday first of February 1836), I saw Mrs. W. in Great Russell-street, and she then only complained of her usual head-aches. She was preparing to entertain a large dinner party, and invited me to join it. I heard no more of this lady, till the above date, when I found her in the most deplorable condition. From having been very embonpoint, she was reduced to a state of comparative emaciation. Her countenance exhibited indescribable distress, and she was unable to utter a single word. She made attempts, indeed, to cloathe her ideas in language; but only produced inarticulate sounds, which I could not understand. Her maid, however, who had lived long with her, could comprehend her meaning tolerably well. Her intellects were in a state of perfect integrity. The loss of speech was the least part of the misfortune. The power of deglutition was so nearly destroyed, that it was with the greatest difficulty she could swallow a tea-spoonful of thin jelly—some of which was ejected from the mouth. She was literally perishing by hunger and thirst, though not feeling the common sensations of either. She was unable to put out or to move the tongue. This organ appeared to be shrivelled and drawn up into knots. The present symptoms had commenced about four or five months previously, and increased in the most gradual manner, till they had acquired their present state of intensity. As sustenance was the most pressing indication, a tube was ordered to inject broth into the stomach, and enemata of the same, with fifteen drops of Battley in each, were directed to be thrown up every eight hours.

*Wednesday, 3d February.* The lavement was tried on Monday evening, and retained. She slept tolerably well that night. On Tuesday it was twice repeated; but she became sick, and vomited much bile. On Wednesday Mr. Blood (her medical attendant) and myself injected about six ounces of beef-tea into the stomach, by means of the stomach-pump. She bore it very well, and was not sick while we remained. She had a natural motion yesterday. I think she appears to have rather more animation in her countenance. She got down a few tea spoonfuls of coffee this morning.

*Saturday 6th February.* The injection into the bowels, and the introduction of some broth into the stomach was practised on Thursday and Friday. They were all retained, though she was once or twice sick, and brought up some bile. She certainly appears rather better to-day, and can produce a slight protrusive motion of the tongue. She sleeps tolerably well, and this morning got down a small quantity of coffee, and a very minute portion of bread and butter, though with great difficulty. She has now great tenderness in the epigastric region, apparently from the distention of the stomach, so long collapsed, and the introduction of broth into an organ which has been unaccustomed to nutriment for so many weeks. A mustard poultice was ordered to the pit of the stomach, and it was suggested that galvanic shocks should be thrown from the back of the head to the front of the larynx, in the course of the eighth and ninth nerves or their branches.

*Saturday, 13th February.* I have visited this lady two or three times since last report. She can receive more liquid nourishment into the stomach than at first, before sickness or attempt at vomiting is made. I may say ATTEMPTS, for she never actually vomits. A very small portion of the last part of the fluid injected, seems to regurgitate from the lower extremity of the œsophagus, but no more. Galvanic shocks were thrown three times, by Mr. Le Beaume, along the nerves. They produced convulsive motions in the tongue and muscles of deglutition. This day (13th February) she could protrude the tongue a little beyond the teeth, and I could understand several words which she attempted to utter. Notwithstanding the nutriment which is injected daily into the stomach and bowels, I think she loses power and flesh. We have now determined to mix equal parts of broth and arrow-root, in order to vary the species of nourishment—the urine is clear, the fæces

(though in small quantities) are natural—the mental faculties unclouded.

*February 19th.* She remains nearly in *statu quo*. The galvanism has done no good. She sleeps much better, however, and retains the broth injections without discomfort. The same cannot be said of the arrow-root and milk thrown into the stomach. It makes her sick, and part of it has lately returned. She still emaciates. There can be little doubt that the pneumogastric nerves are greatly impaired, as far as the stomach is concerned. She evinces no difficulty of breathing. There is no sense of hunger or thirst. *Monday, 22d of February*. She is evidently sinking, and no farther attempts are made by galvanism, or the introduction of fluids into the stomach by the tube. Arrow-root and broth are thrown up daily into the bowels, as they now produce little or no inconvenience. There is no paralysis of any other muscles than those of the tongue and pharynx—not the least aberration of intellectual faculties.

*Sunday, 28th February*, 1836. She sunk in the most gradual manner, and expired this morning, at 1 o'clock, A.M. having been insensible for 24 hours; but able to move her arms till within a few hours of her death.

*Dissection.* The body was examined by Mr. Henry James Johnson, in presence of Mr. Blood, Mr. Bate, and myself.

The vessels of the brain and its membranes were extremely vascular, with some serous effusion between the membranes. The skull was remarkably thick. The substance of the brain was particularly firm. Very little fluid in any of the cavities. The origins of the nerves up to the eighth and ninth pairs were examined, but no lesion could be found. On subverting the brain, the pons varolii and medulla oblongata, together with the superior portion of the medulla spinalis, appeared rather wasted, and their substance somewhat softer than the other parts of the brain. The left vertebral artery, from its emergence from the bony canal to its termination, was dilated to more than the size of the carotid, and thus pressed on the corpus olivare and corpus pyramidale of that side, which appeared smaller than those of the opposite side.* The origins of the left eighth and ninth nerves were consequently pressed upon. The right vertebral artery was remarkably small. On tracing down the eighth pair and the œsophagus through the thorax, we came to an aneurysmal pouch or dilatation of the descending aorta, the size of a large walnut, over which the nervus vagus of that side was stretched in the form of a C. It did not appear that the calibre of the œsophagus itself, was diminished at this place, and therefore the inability to swallow did not arise from mechanical causes. If the pressure on the origins of the nerves had existed, *on both sides* alike, the cause of the paralysis of the organs of speech and swallowing, would have been here incontrovertible; but it may fairly be presumed that such pressure would have been incompatible with life for a single day. The pressure, on one side, may have been sufficient to paralyze the muscles of the tongue and pharynx, without immediately destroying the functions of respiration and digestion, which were carried on by the opposite nerves for a time—but only for a time. The connexion of the phenomena during life with the *post mortem* appearances, is here, probably, as evident as in most other cases where we are accustomed to trace it.

---

### ADVANTAGES OF NEW REMEDIES.

If there are some disagreeable circumstances connected with the performance of the duties of a Journalist, he has peculiar sources of gratification to oppose to them. Scarcely an editorial period passes, but it devolves on him to announce the pleasing intelligence that some incurable disease may be cured. To a philanthropical reviewer

* The vertebral artery was afterwards found enlarged at its *origin*, and the probability is that it was so in the bony canal.

what can be more productive of pure delight, than the spectacle of the miraculous effects of almost all the new remedies? He is able to announce to afflicted mortality, that Dr. A. cures amaurosis, and paralysis dependent on organic changes by strychnine; that Mr. B. removes primary and secondary venereal symptoms utterly opposed in character—lichen—tubercle—and phagedæna—by iodine; that Mr. C. routs scrofula, malignant tumors, rheumatism, and almost every nosological variety of disease by the same powerful yet harmless remedy; that each has his favourite mode of curing tetanus, by medicines so alike in their modus operandi as blisters, tobacco, purgatives, or carbonate of iron; and that every now and then some new drug is thrown up, which, like creosote, may be given in every case with almost equal advantage.

Gratified as an editor must be, he soon learns to be astonished that, when so many specifics exist, any further specifics should be wanted. Incredible as it may appear, each writer would seem to disregard the discoveries of another, and every new marvellous remedy is ushered in, with expressions of regret at the inefficacy of the rest. This is indeed surprising.

Perhaps we may explain so striking a contradiction, by supposing that the profession experiences between these numerous specifics the same feeling of uncertainty as the ass did betwixt the bundles of hay. Doubting which to prefer, he died from absolute starvation. How else are we to account for the still unabated fatality of some diseases, and the obstinate characters of others—fatality and obstinacy that are otherwise irreconcileable with the triumphant success that we daily read of.

It has always been a sort of reproach to physicians, that fashions prevailed in medicine as in coats. If a plausible doctor wrote on the nerves, nervous disorders immediately prevailed. If asthma was the subject of his erudite researches, lo, the whole tribe of spinsters and of dowagers became exceeding short of breath. But now we have another description of mode. Every month enjoys its new remedy, and its patron hands round the *carte* of its virtues.

There is one peculiarity about a new remedy, which distinguishes it pre-eminently from all the old ones. An ancient tonic is not found to possess at once the apparently inconsistent properties of a tonic and a febrifuge. But a modern drug is like old Balderson in the Bride of Lammermuir, who was at once cook, scullion, seneschal, and valet-de-chambre—*it* increases action or arrests it, attenuates or fattens, cures a dropsy, a marasmus, a fever, or a pox, just at the will of the prescriber. It must be owned that this is an important advantage possessed by the new remedies.

It has been thought by some persons, who ought perhaps to have known better, that the cultivation of morbid anatomy would tend to philosophize our experiments with medicines. These short-sighted expectants have materially underrated the effects of two powerful principles in human nature—the love of novelty and the love of noise. Both passions are gratified when their owner appears the successful experimentalist with a novel remedy—the Columbus in the New World of the materia medica.

The patrons of the new remedies would seem, in their experiments, to proceed on the principle of that hospital physician, who ordered his clerk to purge the south ward and vomit the north. They appear to have heard of the observation of the late Dr. Pearson. He was testing the effects of the sulphate of barytes. He gave it to almost every patient under his care. When surprise was expressed at this method of procedure, he replied naively enough —" How can I tell what its effects are, unless I give it in every disease." To be sure, a patient or two may die; but that cannot be balanced against scientific curiosity.

Reasonable as the practice and opinions of these gentlemen may be, there are some individuals who recommend a contrary course of proceeding. Actuated by, perhaps, an improper regard for the rules of logic and the principles of inductive reasoning, they prescribe a course far too methodical and slow for the present day, when every body hastens to introduce himself to the weekly journals. They are so far behind the spirit of the times as to suppose that the following are requisites for conducting a philosophical examination of the properties of a new medicine. They think that :—

1. We should ascertain by appropriate trials, the effects of the drug on the functions, when undisturbed by disease. We should learn, or try to learn, whether the remedy has simply one action on the body or several—whether its effects are altered in kind, or merely affected in intensity, by increase or diminution of dose, or by different states, not incompatible with the health of the recipient. This may be termed our acquaintance with the elementary properties of the drug.

2. Having ascertained its physiological action, we should select those cases for experiment in which such a physiological action is desirable.

3. Applying it in such cases, we should carefully investigate its value as compared with other medicines possessed of similar or analogous properties. Here it is that experimentalists almost always sin. Nine times out of ten the last remedy is vaunted at the expense of others and familiar ones, possessed of equal or superior powers.

4. Pursuing the experiment in the manner we have mentioned, a man of observation has acquired an extensive acquaintance with the powers of the remedy he has been testing. He knows enough, at all events, to obviate the probability of his doing mischief, and now he may carefully experiment in other cases, where reasoning does not point out so obviously the propriety of the attempt. But this, which is the last step of a rational surgeon and physician, is, under ordinary circumstances, the first. Anxious for éclat, the experimenter runs a-muck at every horrid or incurable disease, and in " a little month," indeed much less, out comes the catalogue of marvellous cures, that would make even Katerfelto stare. It is in this sort of experiment that most judgment is required ; on the one hand, we are not to plunge wildly into extravagance, we are not, to " dip our hand in the lucky bag," with the slightest chance in the world of a prize—and, on the other, we must not be trammelled by theoretical considerations, and prevented from attempting to succeed, because, on hypothetical grounds, we ought not.

If there be any truth in the preceding observations, it is not every one who is calculated to investigate the properties of a new medicine. Independently of the mental qualities which we need not dwell on, mental qualities which are obviously requisite, and more requisite than common—independently, we say, of these, the experimenter should be well acquainted with pathology, in order that he may know on what he is experimenting; and he should be practically conversant with the effects of other medicines, in order that he may have a proper standard of comparison. When we add to these necessary ingredients in a philosophical experimentalist — caution in forming conclusions and deliberation in publishing them, we sketch a picture of what the majority of modern experimenters—are not.*

The preceding remarks may be deemed applicable or otherwise to the following recommendation of colchicum, as a remedy for tetanus, just in the ratio of the faith of our readers. For our own parts we grieve to say that we doubt, and we fear that colchicum will prove no match for *acute* tetanus. We say acute tetanus, because chronic tetanus, as well as the milder forms of that malady are well known to yield under many opposite methods of treatment, and, probably, in the main, from the powers of nature. However we will state the observations and experience of Dr. Smith, an American phy-

---

* We are happy to say that we possess some amongst us, who are happy exceptions to the inconsiderate class of persons whom the Reviewer describes. It is not necessary to mention any names.—EDS.

sician, practising at Port-au-Prince. We will insert a portion of his communication on the subject, contained in the American Journal of the Medical Sciences for November of last year.

"In Hayti," says Dr. Smith, "as in all warm climates, tetanus prevails to a considerable extent, but it would seem most confined to the natives, as I have never known a case to occur in an European, or any stranger whatever. It also appears, that persons in this country from the age of five years to thirty, or thirty-five, are most predisposed to attacks of this disease. In the course of little more than two years and a half, nine cases of tetanus have fallen under my observation, and the greater number of these were under my own care. Women do not seem to be so susceptible of tetanus in any of its varieties as men; of nine cases, only one having occurred in a female. Of the nine cases, two were traumatic, and were induced by very slight wounds, the remaining seven were idiopathic, no direct cause being present.

| | |
|---|---|
| Traumatic Trismus. | 2 males, under the age of 20, 1 died, 1 cured. |
| Idiopathic Opisthotonos. | 3 males, under the age of 30, 1 died, 2 cured.<br>1 female, under the age of 40, died.<br>2 males, under the age of 25, cured. |
| Idiopathic Pleurosthotonos. | 1 male, aged 15, died. |

Total, 9—cured 5—deaths, 4.

The four last cases of tetanus which have come under my care, were treated with the Colchicum autumnale, the three out of the four recovered. The following is the general course I pursue, in the treatment of the disease:—On being called to a case of tetanus, my attention is first turned to the actual state of the bowels; constipation in this disease is at least a grave symptom, and should be relieved by injections. I prefer some emollient fluid for this purpose, because I believe that in almost all cases, there is conjoined with costiveness, some degree of intestinal irritation. I commence by administering an enema of ℔j. decoction of either flaxseed or the okra, (Ketmia gombo), cum ol. ricini, ℥iij. or ℥iv. for an adult. After this, fifty or sixty leeches are applied to the spine, from the neck to the sacrum; in my particular practice, I have made choice of the scarificators and cupping-glasses, and the result has been most satisfactory. When the muscles of the jaws and neck are affected, as is most usually the case, leeches are also applied to the mastoid processes; the moment the cups or leeches, whichever may have been used, are removed, cloths wrung out of a strong solution of the muriate of ammonia, are constantly applied to the whole of the vertebral column, the back and the neck. Internally I administer the vinous tinct. of colchicum, commencing with ʒss.; the dose is increased every half hour, and repeated until emesis or catharsis has been produced. As soon as one or other of these effects is obtained, this remedy is to be discontinued; if there afterwards occur any colic or griping, with exhaustion, as in all probability there will, I am in the habit of giving the spt. mindereri ℥ss. every hour, with the addition of one-fourth of a grain of acetate morphine, in solution. If the surface be cold, and there be symptoms of collapse, warm applications are made to the extremities, and the axilla; and the muriated solution is discontinued. The seeds of the colchicum appear more permanent in effect than the bulb; I accordingly make a tincture as follows:—℞. Sem. colch. sicc. ℥ij.; Vin. albi. hisp. ℔j. Infus. &c. as the books recommend.

All the cases of tetanus which have fallen under my care, have been, with two exceptions, treated as above stated, with a very happy result. The exceptions, were the two first cases I treated in this island, and in which I employed opium, general venesection, terebinth. &c. &c.; the patients were put into a warm bath, but, on being taken out of the bath, the spasms became more alarming, the limbs more rigid, and in one instance, death immediately followed."

Dr. Smith appends a case to the preceding observations, but we think we may leave the recommendation of colchicum, as a remedy for tetanus, to the practical portion of our readers. Opportunities of testing its value are unfortunately too frequent.

---

GERMAN CONTRIBUTIONS TO PRACTICAL MEDICINE AND SURGERY, CHIEFLY DERIVED FROM RUST'S MAGAZINE FUR DIE GESAMMTE HEILKUNDE, AND COMMUNICATED BY ROBERT THACKER, M.D. Derby.

*Polypus Uteri within the Womb successfully removed by Ligature.*

This is one of three interesting and instructive cases of Polypus Uteri related by Dr. Buck, of Lübeck. The object of the narrator is to shew how erroneous is the opinion generally—we may almost say—universally received by surgeons, that morbid growths of this nature, arising from the fundus of the uterus, do not admit of removal as long as they are contained within the cavity of that organ. "Experience has taught me," says the Doctor, "that when the *os tincæ* is open, it is practicable to apply a ligature to a polypus within the uterus; and that this operation is always indicated whenever an inactive state of the organ renders it incapable of effecting the protrusion of its contents, and at the same time the constitutional disturbance threatens imminent danger to the life of the patient." Such a contingency, indeed, does not often present itself; and the operation in question is no doubt one which it will but seldom be necessary to have recourse to. That this necessity does, however, sometimes occur, the following case abundantly proves, and we may add, that it reflects not a little credit on Dr. Buck, who, undismayed by the known difficulties, and the supposed impracticability, of the operation, nevertheless undertook it, performed it with success, and thereby saved his patient's life. We shall extract the case at length.

"In January, 1834, I was called to see a poor woman, who gave me the following account of her sufferings:—She was 46 years old, had previously enjoyed good health, and was the mother of six children. Since the death of her husband, which took place nine years before, she had abstained from sexual intercourse. Two years previously, she was seized, in place of the ordinary menstrual discharge, with a violent hæmorrhage, which ceased after a duration of eight days, and for the space of a year did not return. In this interval she began to suffer from an oppressive sense of weight in the belly, pricking pains in the side and in the pelvis, difficulty in voiding urine, and a dragging sensation in the sacral and lumbar regions.

These symptoms gradually increased in severity, and about Whitsuntide, 1833, hæmorrhage from the uterus again took place, and continued to recur at short intervals until about six weeks before the period of my visit, when it ceased altogether, and a fluor albus, having an offensive smell, appeared in its place. I found the patient in a state of extreme emaciation, cachectic in her appearance, and so weak as to be scarce able to leave her bed. She complained of violent pains in the iliac region, which were augmented by pressure, and from time to time of slight pains resembling those of labour; her appetite was bad, her pulse small and quick, she coughed a good deal, and vomited occasionally.

On making a manual investigation, I found the uterus in its normal situation, the lips thin and flaccid, and the os uteri itself open, so that it admitted the index finger with facility. Within the organ, and apparently arising from its fundus, a polypus was clearly to be felt; it had the circumference of a large pear, was insensible to moderate pressure, and did not quite reach to the external orifice of the uterus. The parts were covered with a mucous exudation streaked with blood, and of an offensive smell.

I directed a plan of treatment calculated to improve her general health, in the hope that the labour-like pains would at length expel the polypus from the cavity of the uterus. Deceived in this expectation, I prescribed 24 powders, each of which contained half a scruple of secale cornutum, and these failing to produce any effect, nine others containing one scruple each; the latter were all taken in 36 hours; nevertheless, the uterus still remained in a state of inactivity, and even the stomach, an injurious action upon which has been attributed to this medicine, was not in any way affected by it.

Dreading the difficulty of applying a ligature to a polypus so situated, and as there were no dangerous hæmorrhages, I still waited, hoping that the powers of nature would finally effect the desired protrusion. But the health of the patient daily grew worse, the mucous secretion became more and more copious, and the cough, which had hitherto been dry, was now accompanied by an abundant and ill-looking expectoration, together with pains in the breast. Frequent though slight febrile paroxysms, nocturnal sweats, and a watery diarrhœa, added to the prejudicial influence of many privations, and the want of proper attendance, soon reduced my unfortunate patient to a state of complete marasmus, so that I already believed her end near, and certainly under these circumstances should have desisted from all operative proceeding, had I not convinced myself from previous experience, that now, the speedy removal of the polypus alone afforded a chance of recovery.

With the assistance, therefore, of Dr. Pabst, I undertook to apply a ligature round the root of the polypus, selecting for this purpose Jörg's double canula.* The patient being placed upon a cross-bed in a half-sitting position, I introduced the instrument previously oiled, and provided with a thread, upon the left index finger, and pushing it carefully through the os tincæ, between the polypus and the yielding uterine parietes, carried it as near the root of the tumor as possible. One canula being firmly held in this position, I at length succeeded, after many fruitless attempts, in passing the other one, with its concavity always turned towards the polypus, fairly round the pedicle, and I now joined the two together. The pain occasioned by the unavoidable friction on the inner surface of the uterus, was trifling, nor did this stimulus provoke the organ to the slightest contractile effort. On tightening the thread, I was convinced by the insensibility of the parts, that I had not included any portion of the uterus, a circumstance the more to be dreaded as the point where the ligature was applied, was quite out of the reach of the finger. During eight days, I daily drew the thread tighter, it then broke, and on examination I discovered that only half of the polypus had sphacelated away. A re-application of the ligature was successfully effected, though not without considerable difficulty, as the body of the tumor had much diminished in size and firmness, while the pedicle seemed proportionally thick and sinewy. In the mean time, the injurious influence of the copious and highly fetid discharge was, as far as possible, counteracted by the frequent use of antiseptic injections.

In the course of a few days, the last ligature came away, and at the end of a fortnight no remains of the polypus were discoverable; the uterus, too, had contracted, and the os tincæ soon after assumed its normal form.

The patient, who had hitherto been lying in an almost hopeless state, now recovered strength surprisingly fast; the diarrhœa, fever, nocturnal sweats, and puriform expectoration, quickly disappeared, and at the expiration of two months, with the aid of tonic medicines, she was restored to perfect health."

* I chose this instrument on account of its length, but found that the curvature at its upper end rendered the operation more difficult, and I now employ in preference two straight silver canulas which separate and unite with greater ease.

*Vaginal Hernia of the Bladder, the Diagnosis presenting unusual Difficulties.*

This is an extremely curious case, and one pregnant with instruction. It is related by Dr. Buck, of Lübeck, the same on whom we lately had occasion to pass a merited eulogium, but who now appears before us under a more questionable shape. It will be seen that he committed a great blunder in his diagnosis, which nearly led to very disastrous consequences, neither more nor less, indeed, than a summary excision of nearly the whole of the bladder. He, however, honestly confesses his error; and certainly there are few who would not have been misled by the remarkable combination of deceptive appearances which the following case presented.

"A woman, ætat. 54, of seemingly sound constitution, 12 years previously had borne her second and last child. The labour was long and tedious, and the placenta, which, according to the statement of the midwife, was adherent to the uterus, was brought away by the hand. From this period she frequently experienced a sudden sensation of emptiness in the abdomen, from which something appeared, as it were, to sink down, and at the same time a feeling of fullness and pressure in the vagina, all which symptoms soon disappeared on assuming the recumbent posture. Their character, however, became progressively more marked, and one day she discovered, projecting into the vagina, a round foreign body, which perceptibly increased in size, particularly after corporeal exertion.

Her situation as childrens' nurse in St. Ann's poor-house, not requiring hard labour, it was only after a lapse of some years that the tumour had so augmented as to protrude out of the vagina, and at that time, by abstaining for a while from all exercise, the patient was enabled to reduce it herself. She did not seek medical advice, and for many years, during which the tumour continued to increase in size and the protrusions in frequency, she always succeeded in replacing the body without assistance. At length, having descended lower than usual, it obstinately resisted all her attempts at reposition, and now remained stationary, forming a large and painful swelling projecting through the vulva. At no time had she experienced difficulty in passing her urine. For a quarter of a year she bore her sufferings in silence, still continuing to attend to her duties: and it was only when become incapable of quitting her bed, that she was induced to apply for medical assistance.

On examination, I found before the labia majora a hard body, equal in size to the head of a seven-months' fœtus, no where yielding to pressure, and covered in its greater part by cauliflower excrescences, which secreted an ill-smelling ichor; its lower third was not yet attacked by the ulcerative process, was of a vivid red colour, and presented very accurately, only on a larger scale, the form of the vaginal portion of the uterus. At the extremity of this part, a transverse slit regarded by us as the os uteri, admitted a probe to the depth of half an inch. The body of the tumour appeared closely invested by the inverted and protruded vagina, and its base had contracted firm adhesions with the inner membrane of the labia majora, so that inferiorly it was continuous, and formed a straight line with the perineum. On examination per rectum, the uterus was no where to be felt. I made many attempts to introduce the catheter in the usual way, but in this was constantly foiled, the urethral orifice being concealed by large and painful excrescences, which bled on the slightest touch. However, the urine was voided without difficulty, and in normal quantity, the only inconvenience being that resulting from the unavoidable wetting of the surrounding ulcerated surfaces: the evacuation was not followed by any perceptible alteration in the bulk of the tumour, nor did pressure upon the latter at any time occasion a desire to make water.

The state of the patient's health was as yet tolerable, although frequent slight attacks of fever, and much dejection of mind, made it sufficiently clear that the disease was beginning to act injuriously on the constitution.

I considered the case to be one of complete prolapsus of the uterus, which from exposure to many causes of irritation, in particular the air, the urine, and the frequent ill-directed efforts at reposition, had been attacked by a slow inflammation, which at length had passed into cancerous ulceration.

In order to liberate the patient from a disease which must, else, inevitably prove fatal, I determined to extirpate the cancerous uterus, a proceeding which I decided to adopt not only because it is a surgical aphorism that in such cases this operation presents the only chance of preserving life, but also from a consideration of the following circumstances :—the age of the patient, who had passed the grand climacteric, the manner in which the disease originated, viz. by external injuries alone, the completeness of the protrusion, and, finally, the still tolerable state of the general health. In these views of the nature of the disease, and of the plan which it was necessary to adopt, I was supported by many of my colleagues, practising surgeons and accoucheurs, who had frequently seen and examined the patient.

It was my intention to dissect the inverted vagina from the uterus, and then to remove the latter by separating its remaining connexions, at the same time carefully avoiding the adjacent parts.

The rectum having been emptied, the patient was placed upon the operation table, and fixed in the same position as in the operation for lithotomy. After many fruitless attempts by different individuals, who all acted under the supposition that the direction of the urethra was normal, Dr. M. at length succeeded in introducing the catheter into the bladder by holding the instrument almost perpendicularly, which would not have been possible had the bladder been in its natural position. As we were unable to feel the extremity of the catheter through the protruded body, but by examination through the rectum, distinctly discovered it at the posterior part of the swelling, we concluded, although feeling somewhat insccure with regard to our diagnosis, that the bladder had sunk down behind the prolapsed uterus, and rested against the posterior surface of that organ ; a supposition which seemed to explain satisfactorily the perpendicular direction of the urethra. Notwithstanding this discovery, it was unanimously deemed necessaiy to proceed with the operation.

The tumour being a little raised, I made an incision several inches in length along its inferior surface, and commenced dissecting away the thickened vagina, carefully avoiding the adjoining rectum by turning the edge of the bistoury towards the tumour. But to my astonishment, instead of exposing the substance of the uterus, I came upon true muscular fibres, and distinctly felt the catheter through the part from which I had removed the dense vaginal investment. These two circumstances convinced us, that the greater portion of the tumour was nothing else than the prolapsed bladder, but we were still unable to decide on the relative situation of the uterus, and whether, in fact, this organ formed part of the tumour. From the urethral orifice where the most considerable excrescences existed, I now made a sufficiently deep incision, united this with the inferior one, and thus dissected off the whole leather-like envelope, together with the fungous masses. The denuded bladder was in no place wounded, and it had lost so much of its hardness, although its parietes were still unusually thick and dense, that the catheter was easily discoverable at every part of its surface. The operation was rendered more difficult by a pretty severe hæmorrhage, which, however, after tying a few small vessels, soon yielded to cold affusions. The bulk of the swelling was now diminished by nearly one half, the surface was smooth and freed from cauliflower excrescences, and after some gentle attempts at reposition, it sunk a little back spontaneously. The patient, after taking a laudanum draught, was carried to bed.

The first days following the operation, with the exception of some degree of febrile excitement, her state on the whole was as favourable as could be expected : the appetite was tolerable, her nights were passed quietly, she experienced but little pain in the parts operated upon, and none in the abdomen, which was also free from tympanitic distention. The parts were at first bathed with cold water, afterwards gently stimulating fomentations were employed. The urine, unaltered in colour, was drawn off from time to time by a catheter, which was retained constantly in the bladder. For several days the bowels acted naturally, but at a later period it was found necessary to administer an occasional enema. The prolapsed bladder continued to recede gradually, and the granulating surface presented a kindly aspect, and secreted healthy pus. Such was the state of our patient during three weeks and a half, and sanguine hopes were entertained of her eventual recovery; but she now gradually lost her appetite and sleep, emaciation commenced, and, attended by a slow fever, proceeded with rapid strides, the surface of the wound became dry and red, and a colliquative diarrhœa, which nothing could restrain, finally carried her off six weeks after the operation.

*Autopsy.* The body was extremely emaciated, and on the inner side of the left arm, we observed a lipomatous tumour of considerable size. The organs of generation, together with the bladder and a portion of the pelvis, having been carefully removed, the following facts were ascertained: the entire protruded body, which we at first believed to be the uterus, was a prolapsus of that part of the bladder uncovered by peritoneum, and which, in its gradual descent, had pushed before it the upper wall of the vagina. The lower third of the tumour, so closely simulating in form the vaginal portion of the uterus, was free from scirrhous degeneration, and formed a compact mass, superficially smooth, and of an almost cartilaginous consistence. An incision made into this part from below upwards, only reached the bladder at the depth of one inch and a half. The slit at its extremity, mistaken for the os uteri, and which had contributed to our error more than anything else, was formed by a peculiar duplicature of the vagina, the origin of which it is difficult to explain. Worthy of remark in this case was the extraordinary dilation undergone by the vagina. For the upper wall, with which the bladder is naturally in contact, could alone have formed the bag by which the tumour was invested: the remainder of the vagina anteriorly intimately united with the bladder, was continued up to the uterus as in the normal state. That the vesical hernia did not take place through a rupture of the vagina, was rendered evident by the thick leathery envelope of the tumour, as well as by the uniform absence of those symptoms which would necessarily have attended an occurrence of this nature. The coats of the protruded portion of the viscus were in a thickened degenerated state; those of the part covered by peritoneum, and which was still within the cavity of the pelvis, had suffered little alteration. The uterus itself was found lying in the concavity of the sacrum, in a perfectly healthy state, only somewhat higher in the pelvis than is usual.

Such is the history of this remarkable case, and when we regard the external appearances, in particular the deceptive resemblance of the hard and symmetrical extremity of the tumour to the vaginal portion of the uterus, and also the total absence of all symptoms indicating a lesion of the bladder, we cannot but believe ourselves justified in having mistaken the nature of the disease even until the eleventh hour. Had our diagnosis been correct, there must still have been a necessity for the operation, by which the cancerous and degenerated vagina was alone removed, without injury to the bladder or the surrounding structures."

The two following cases we select from a paper by Dr. Creutzwieser, a physician practising at Königsberg in Prussia. The first is brought forward by the author to shew—though, indeed, on this side of the German Ocean we are tolerably well acquainted with the fact—that in all compound fractures of the skull, it is not absolutely indispensable that the patient be forthwith trepanned. In the case under consideration, Dr. Creutzwieser did not trepan only because he was not permitted: the man nevertheless recovered: query, had the operation been performed, would the case have then terminated favourably? We think that an English surgeon, under similar circumstances, would not have deemed such a proceeding necessary; but our readers shall judge for themselves.

### *Compound Fracture of the Skull, cured without Trepanning.*

"Michael Wardowsky, a forester, aged 45 years, of a sound and robust constitution, went one morning to cut wood, accompanied by his wife, and having mounted a beech, and seated himself across one of the largest branches, the simple fellow, emulous, it should seem, of an exploit which has been performed more than once, commenced hacking with great vigour at that part of the bough between himself and the trunk of the tree! In due course of time the bough broke, and Wardowsky was precipitated backwards: he fell from a considerable height upon a waggon which chanced to be standing beneath, laden with dry wood, and alighting on the back of the head, the scalp at this part was torn up from its connexions to the extent of more than two inches. His wife, who was at work near the scene of the accident, hastened to him, bound a cloth moistened with urine firmly round his head, and led him carefully home. On his arrival, he was seized with continued vomiting, raved violently, tore the dressings from his head, and required the united strength of several men to hold him. He was at length securely bound down, and six hours afterwards I visited him.

On examining the wound, which bled freely, and ran from right to left obliquely across the occiput and over a portion of both parietal bones, I soon discovered a fracture of the left *os parietale*; it had the form of an irregular cross, and was about the size of a Prussian achthalber. Nothing could be clearer—here was a case for the trepan: but to this the wife and brother of the patient were resolutely opposed; it was in vain that I urged the necessity; they were not to be moved, and I was therefore obliged to give up the point. The fractured part was not depressed. The face being flushed and swollen, and the pulse full and frequent, I took 12 ounces of blood from the arm, and applied 20 leeches to the forehead and nape of the neck; I also directed the feet and legs to be placed in a hot mustard bath, and a clyster, composed of vinegar and decoction of chamomile, to be administered every hour. The following medicine was prescribed:—

℞. Nitr. depur. ʒij.

Aq. distill. ℥v.

Natr. sulphuric, ℥ss.

Aq. sauroceras. ʒj. M. Capiat æger cochleare unum omni semihorâ.

The separated portion of the scalp was retained in its place by a few strips of adhesive plaster, and a bladder filled with ice placed over the injured parts. In the course of a few hours the face became pale, the pulse slow, and the intellect clear; the bowels acted by a voluntary impulse, the vomiting ceased, and the patient fell into a quiet sleep. By persevering in the above treatment, and observing a very severe regimen, the second, third, and fourth days were passed by the patient in this favourable state: but at this period he suddenly became delirious, violent febrile symptoms presented themselves, the eyes sparkled, the urine was clear as water. An inflammation of the brain had supervened, and the game appeared lost. I immediately abstracted 12 ounces of blood, applied, as before, 20 leeches to the forehead, and directed a grain of calomel to be given every hour. After 24 grains of this medicine had been taken, and the venesection repeated to the amount of eight ounces, these threatening symptoms, contrary to expectation, entirely vanished, and within three weeks the patient was able to leave his bed.

In six weeks the cure was completed, and at the present time, two years after the period of the accident, Wardowsky still lives, and enjoys his usual good health."

*Hydrocephalus Chronicus Externus.*

On this case of chronic hydrocephalus, to which we have already alluded, we shall make no other remark than, that a more extraordinary one never fell under our notice.

"N. N., the illegitimate son of a poor woman, is now 26 years old.—The mother states that, at his birth, the head was soft, pulpy and unusually large, and that in his third year the fontanels were not yet closed. The growth of the child was slow, the period of teething occurred later than usual, and he was longer in learning to walk and to speak than children generally are. Not thus tardy was the development of the intellectual faculties, for, at the age of four years, the boy already gave evidence that he possessed a quick perception and a sound understanding. As he grew older, the organic disproportion between the head and the rest of the body became constantly more and more marked; and the deformity has at length attained a degree which, together with other morbid changes, I will now attempt to describe.

At that part of the head which corresponds to the junction of the sagittal and lambdoid sutures, there exists a depression, half an inch deep, and an inch in diameter: it is lined by the integuments and the hair of the head, and admits the half of a hen's egg, placed in the cavity with its more obtuse extremity downwards: the said egg is then observed to rise and fall synchronously with the expirations and inspirations. The entire left half of the face is normal and well formed, but the right is misshapen in all its parts, insomuch that the eye, the half of the nose, the ear, the half of the mouth, and the cheek are half an inch lower than the corresponding features of the opposite side, and their organic substance so much increased that the right side of the face presents the appearance of a large and elastic fleshy bag filled with water. If this pocket-shaped cheek is somewhat suddenly pressed upwards, the hole at the upper and back part of the head is instantly filled up, and the object placed within it, an egg or an apple, thrown out! However strange this may sound, it is nevertheless true, and on a closer examination of the head, the phenomenon appears to be accounted for. It is then observed that the zygomatic process* of the *os frontis* is not in contact with the malar bone, but separated from it by a space which might admit a goose-quill; and farther, that a similar slit exists in the mastoid process of the temporal bone of the same side. Still more striking is the defect of formation in the inferior maxillary bone: it is divided into three parts, distinct from each other, and only held in apposition by the buccinators and the muscles of the face and neck. In each of these divisions are two sound teeth, fixed in equally sound alveoli, and during mastication, as may be supposed, the face of this unfortunate being suffers very strange contortions. The experiment already described proves the connexion of the water collected in the cheek with that in the cavity of the skull, and this connexion is no doubt effected through the medium of the above singular slits in the bone. At first I was inclined to believe that the fluid was situated immediately beneath the common integuments, and had no relation to the cerebral cavity, but the following experiment convinced me of the error of this supposition: I applied a bandage tightly round the head, so as to cut off all communication between the face and the parts external to the skull, and now observed that by pressing as before upon the right cheek, an instantaneous filling up of the cavity on the head nevertheless took place: and it is also to be remarked that this experiment always produced a transient giddiness.

The deformity of the face is still increasing, and the patient will soon find it necessary to support his protuberant cheek by a suspensory bag. His general health is not at all impaired, nor are his mental faculties in any degree weakened.

---

* The external angular process of English anatomists.—*Tr.*

A post-mortem examination will one day afford a more accurate explanation of this anomalous case.

---

### Paralysis Crescens ab imo.

An interesting case of this kind lately occurred in the practice of Dr. Johnson. A gentleman, now about 28 years of age, experienced an epileptic seizure nearly seven years ago, while under some violent excitement. In the course of the next five years he had, at very unequal epochs, four attacks of the same kind—all under circumstances of moral or physical excitement. One interval was upwards of two years. The last seizure was slight, and occurred in the beginning of 1834. Since that period, a new train of phenomena took place. He first began to perceive a diminution of power and sensation in his toes—then of the ankles—then of the knees, causing him to walk with tottering steps. When the paralysis had advanced thus far, the fingers became a little benumbed and less powerful—then the wrists—and now (April 1836) he cannot write, with much degree of legibility—nor assist himself by means of his hands or arms. Three or four months ago, the power of the bladder and rectum began to diminish. He is now quite incapable of expelling any urine, except by the action of the abdominal muscles, when he goes to stool, which is regularly once in 24 hours. Urine and fæces occasionally escape involuntarily. All his intellectual powers are in vigour. His memory is more retentive than it was seven years ago, prior to the epileptic attacks. His digestion is yet unimpaired. The functions of circulation, respiration, secretion, &c. perfectly natural. He got married about a year ago, to a young and beautiful lady. The powers of virility have diminished.

A great variety of remedies—or rather medicines, has been tried, without the slightest benefit. Amongst others, strychnine was exhibited, till painful twitchings were experienced in the extremities. A seton was inserted in the nucha—baths of various kinds were used—but all to no purpose.

He came to London, from a great distance, to consult Sir Astley Cooper and Dr. Johnson. A most patient investigation, separately and in consultation, was had, and the joint opinion of the two consultants was—that some organic lesion existed in the spinal column—perhaps in the posterior part of the brain. The precise nature of this lesion, the consultants could not determine. It might be ramollissement—serous effusion within the membranes—or other disease not known. The prognosis was very unfavourable. The remedies were recommended without much hope of cure. They consisted chiefly in counter-irritation to the spine—small doses of the oxymuriate of mercury—and the daily use of the catheter.

---

### Medical Statistics.

Statistical facts in medicine are beginning to assume the importance due to accurate generalizations. They are not yet sufficiently extensive or correct to enable us to ground any positive opinions on the data that they furnish, but already they have dispelled some long-prevailing errors, and established some important laws. We will throw together some observations upon the comparative mortality of males and females, of the married and the unmarried, that have lately appeared in the contemporary journals.

The first paper to which we shall allude is contained in the American Journal of the Medical Sciences, for November 1835. It relates to the comparative mortality of males and females under the age of puberty, and to the actual causes of the difference that obtains.

1. *Observations upon the Mortality in Philadelphia under the Age of Puberty, shewing the excessive Proportion of the Male over the Female Deaths, and the particular Sources from whence it proceeds.* By G. Emerson, M.D.

It appears, from the returns made to the Board of Health of Philadelphia, that during the ten years included between 1821 and 1830, 64,642 children were born there. The excess of males over females was 2,496, or 7½ per cent. The census of 1830 shewed that, by the fifth year of childhood, the excess was reduced to 5 per cent.; and, by the tenth year to 1 per cent.; while, between the ages of ten and fifteen, the females are found to exceed the males by about 8 per cent.; and, beween fifteen and twenty, by 7.3 per cent.

These curious facts are more or less in accordance with what has been observed in other countries. In Europe, as in America, and probably throughout the world, more males than females are born, yet, after the lapse of a certain period, more females than males survive.

Many conjectures respecting the causes of this see-saw of births and deaths, have been hazarded by statistic writers. But conjectures are idle in determining questions which admit of an appeal to positive facts. Dr. Emerson, discarding speculation, has investigated the bills of mortality, for the real causes of the deaths of males, and the special maladies which are most destructive to either sex in early years. The result of his investigations is contained in three Tables. In the first, we are presented with:—The mortality under the twentieth year, from the most frequent sources, during the years 1832, 1833, and 1834, with the numbers of each sex from each disease, and the ratio in which the deaths of the one exceed those of the other sex.

TABLE I.

| DISEASES. | Males. | Females. | Excess. | | Ratio of Excess. |
|---|---|---|---|---|---|
| | | | M. | F. | |
| Consumption | 153 | 185 | | 32 | 17.3 p. ct. |
| Convulsions | 433 | 357 | 76 | | 17.5 |
| Bowel complaints of all kinds, (excepting Cholera Maligna) | 699 | 597 | 101 | | 14.5 |
| Small-pox | 133 | 114 | 19 | | 14.3 |
| Scarlet Fever | 216 | 220 | | 4 | 1.8 |
| Croup | 157 | 120 | 37 | | 23.5 |
| Hooping Cough | 78 | 80 | | 2 | 2.5 |
| Bronchitis | 114 | 84 | 30 | | 26.3 |
| Inflammation of the Brain | 101 | 67 | 34 | | 33.6 |
| of the Lungs | 190 | 151 | 39 | | 20.5 |
| of the Bowels | 184 | 98 | 36 | | 26.8 |
| Fevers of all kinds, (Scarlet excepted) | 185 | 141 | 44 | | 23.7 |
| Dropsy, (general) | 42 | 35 | 7 | | 16.6 |
| of the Head | 288 | 258 | 30 | | 10.4 |
| of the Chest | 18 | 26 | | 8 | 30.07 |
| Casualties | 15 | 8 | 7 | | 46.6 |
| Debility and Decay | 251 | 197 | 54 | | 21.5 |
| Atrophy | 65 | 38 | 27 | | 41.5 |
| Teething | 17 | 16 | 1 | | 5.8 |
| Burns and Scalds | 26 | 35 | | 9 | 25.7 |
| | 3315 | 2827 | 542 | 55 | |
| | 2827 | | 55 | | |
| Total mortality of both sexes from the above diseases | 6142 | | 487 | | |

The male mortality exceeds the female in the ratio of 7.94 per cent. The diseases which seem particularly fatal to the males, are—

Inflammation of the brain, inflammation of the bowels, bronchitis, croup, inflammation of the lungs, fevers of all kinds, (except scarlet,) convulsions, general dropsy, dropsy of the head, small-pox.

To these sources of mortality may be added those under the head of casualties, with others vaguely designated debility, decay, &c.

The few cases in which the deaths of females predominate, are under the following heads :—

Consumption, dropsy of the chest, scarlet fever, burns and scalds, hooping cough.

To shew that the same causes operate with tolerable equality in different years, Dr. Emerson adds the second Table.

TABLE II.

*Infantile Mortality in Philadelphia in the Years* 1832, 1833, *and* 1834, *from the most predominant Causes, with the Numbers of each Sex for the different Years.*

| DISEASES. | 1832. | | 1833. | | 1834. | |
|---|---|---|---|---|---|---|
| | Males. | Females. | Males. | Females. | Males. | Females. |
| Consumption.. .. .. .. .. .. .. .. | 58 | 74 | 54 | 50 | 41 | 61 |
| Convulsions .. .. .. .. .. .. .. .. | 160 | 147 | 130 | 96 | 143 | 114 |
| Bowel complaints, (Cholera Maligna excepted) .. | 299 | 235 | 126 | 132 | 267 | 225 |
| Diarrhœa alone .. .. .. .. .. .. .. .. | 60 | 53 | 21 | 25 | 33 | 40 |
| Small-pox .. .. .. .. .. .. .. .. .. | 8 | 5 | 56 | 52 | 69 | 57 |
| Scarlet Fever .. .. .. .. .. .. .. .. | 150 | 147 | 30 | 31 | 36 | 42 |
| Croup .. .. .. .. .. .. .. .. .. .. | 60 | 47 | 50 | 41 | 47 | 32 |
| Hooping Cough .. .. .. .. .. .. .. .. | 26 | 32 | 26 | 26 | 26 | 22 |
| Bronchitis .. .. .. .. .. .. .. .. .. | 40 | 30 | 15 | 11 | 59 | 43 |
| Inflammations of all kinds .. .. .. .. .. | 197 | 139 | 128 | 100 | 160 | 132 |
| of the Brain.. .. .. .. .. | 34 | 24 | 22 | 14 | 45 | 29 |
| of the Lungs .. .. .. .. | 83 | 60 | 55 | 35 | 52 | 56 |
| of the Bowels .. .. .. .. | 54 | 32 | 39 | 40 | 41 | 26 |
| Fevers of all kinds, (Scarlet excepted) .. .. | 86 | 72 | 59 | 42 | 40 | 27 |
| Dropsy, (general) .. .. .. .. .. .. .. .. | 60 | 53 | 7 | 4 | 16 | 12 |
| of the Head .. .. .. .. .. .. | 102 | 78 | 87 | 83 | 99 | 97 |
| of the Chest .. .. .. .. .. .. | 10 | 12 | 4 | 6 | 4 | 8 |
| Casualties .. .. .. .. .. .. .. .. .. | 5 | 6 | 3 | 2 | 7 | |
| Debility and Decay .. .. .. .. .. .. .. | 95 | 70 | 76 | 69 | 80 | 58 |
| Atrophy.. .. .. .. .. .. .. .. .. .. | | | 11 | 6 | 54 | 32 |
| Teething .. .. .. .. .. .. .. .. .. | 11 | 3 | 3 | 6 | 3 | 7 |
| Burns and Scalds .. .. .. .. .. .. .. .. | 6 | 10 | 11 | 12 | 9 | 13 |

The object of the third Table is different from that of the preceding. It is intended to illustrate the comparative mortalities of various ages under the twentieth year, independently of reference to sex.

TABLE III.

*Infantile Deaths in Philadelphia during the Years* 1832, 1833, *and* 1834, *from the most Common Sources of Mortality, distributed under the various Periods of Life, from the First to the Twentieth Year.*

| DISEASES. | Under 1 year. | From 1 to 2 years. | From 3 to 5. | From 5 to 10. | From 10 to 15. | From 15 to 20 |
|---|---|---|---|---|---|---|
| Consumption.. .. .. .. .. .. .. .. .. | 74 | 48 | 54 | 32 | 26 | 104 |
| Convulsions .. .. .. .. .. .. .. .. .. | 550 | 107 | 94 | 24 | 10 | 5 |
| Cholera Morbus .. .. .. .. .. .. .. .. | 1 | 2 | 6 | 9 | 2 | 3 |
| Maligna .. .. .. .. .. .. .. .. | 4 | 6 | 37 | 42 | 22 | 25 |
| Infantum.. .. .. .. .. .. .. .. | 608 | 266 | 57 | 7 | 2 | 0 |
| Diarrhœa .. .. .. .. .. .. .. .. | 115 | 61 | 32 | 16 | 4 | 4 |
| Dysentery .. .. .. .. .. .. .. .. | 25 | 24 | 17 | 15 | 6 | 2 |
| Small-pox .. .. .. .. .. .. .. .. | 82 | 34 | 76 | 30 | 12 | 13 |
| Scarlet Fever.. .. .. .. .. .. .. .. .. | 33 | 83 | 205 | 100 | 12 | 3 |
| Croup .. .. .. .. .. .. .. .. .. .. | 108 | 63 | 85 | 19 | 2 | 0 |
| Hooping Cough .. .. .. .. .. .. .. .. | 81 | 31 | 37 | 8 | 1 | 1 |
| Bronchitis .. .. .. .. .. .. .. .. .. | 104 | 39 | 36 | 16 | 0 | 3 |
| Inflammation of the Brain .. .. .. .. .. .. | 51 | 36 | 36 | 20 | 13 | 12 |
| of the Lungs .. .. .. .. .. .. | 147 | 82 | 64 | 18 | 7 | 11 |
| of the Bowels and Stomach.. .. | 103 | 37 | 39 | 21 | 12 | 20 |
| Fevers of all kinds, (Puerperal and Scarlet excepted) .. .. .. .. .. .. .. .. .. | 87 | 44 | 70 | 45 | 39 | 45 |
| Dropsy, (general).. .. .. .. .. .. .. .. | 10 | 7 | 21 | 20 | 7 | 12 |
| of the Head .. .. .. .. .. .. .. | 198 | 166 | 116 | 54 | 9 | 3 |
| of the Chest .. .. .. .. .. .. .. | 8 | 4 | 15 | 5 | 6 | 6 |
| Casualties .. .. .. .. .. .. .. .. .. | 1 | 3 | 6 | 4 | 5 | 4 |
| Debility and Decay .. .. .. .. .. .. .. | 329 | 22 | 19 | 6 | 1 | 4 |
| Atrophy and Marasmus .. .. .. .. .. .. | 132 | 71 | 32 | 6 | 2 | 3 |
| Teething.. .. .. .. .. .. .. .. .. .. | 20 | 11 | 2 | 0 | 0 | 0 |
| Burns and Scalds .. .. .. .. .. .. .. .. | 6 | 14 | 22 | 12 | 7 | 4 |
| | 2877 | 1261 | 1178 | 529 | 200 | 287 |
| Of other affections .. .. .. .. .. .. .. .. | 665 | 199 | 217 | 106 | 57 | 92 |
| Total from all sources .. .. .. .. .. .. | 3542 | 1460 | 1395 | 635 | 257 | 379 |

A glance at this Table supports the deduction which has been already drawn from other sources, that mortality diminishes, and that with tolerable regularity, from the first year to the age of puberty, and then, for a few years, again increases. Further comments are, we think, unnecessary. We hope Dr. Emerson will prosecute his inquiries, and lay the results before the profession, so soon as they are sufficiently accurate and extensive to lead to any generalizations of consequence.

2. *Comparative Mortality of Male and Female Life.*

Mr. Rickman has been publishing some elaborate remarks on this, as indeed on other subjects connected with medical statistics, in the Medical Gazette. We shall notice some of the general results which Mr. Rickman draws, or which may be drawn from the data that he furnishes. Mr. Rickman deserves great credit for the industry he has evinced in collecting materials, and the zeal he has displayed in communicating his observations and conclusions to the public.

It is not necessary for us to repeat that the longevity of females exceeds that of males. This was first systematically shewn by Keraboon in a treatise published in the year 1742, and founded on the mortality of government annuitants in Holland. It is not then to prove this fact, already fully proven, that Mr. Rickman writes or that we condense his observations. His object is to shew the various degrees of female longevity in the various classes.

He exhibits several tables. In the first are shewn the results of a tontine, established under "the Million Act," in the year 1695; the last of the 1002 annuitants under which died in the year 1783. In the second table are the results of various tontines and other government annuities, current from 1785 to 1825, the rate of mortality for forty years being displayed. In the next table are the Parish Register Returns of the ages of those buried in eighteen years, to the extent of nearly four millions of persons. And finally we have a table of 1800 burials in Carlisle, between the years 1779 and 1787; and one of the Equitable Life Assurance Society, containing the ages at death of 5144 persons in 67 years, that is, from 1762 to 1829. These tables we need not insert, as their principal value must be to those engaged in actual calculations. We therefore proceed to Mr. Rickman's conclusions.

From the accurate investigation of the ages at death of persons in England *of all classes*, for eight or nine years before, as well as after, the year 1821, it appears that the advantage in favour of the life of females amounts to four per cent. But in the classes who purchase government annuities, classes necessarily above the reach of hardship and privations, the advantage of the female is actually as high as thirteen per cent. A remarkable fact. Mr. Rickman observes in reference to this circumstance :—

"That the increase of civilization should have protected the female sex from hardships of all kinds, especially from exposure to inclement weather, and that this advantage added to their habitual estrangement from excesses in diet and exercise, should have lengthened life ten per cent. beyond that of the male sex, is not incredible, nor very surprising; but that this advantage (for long life is but another word for comfortable life) should have been increased to thirteen per cent. by the progressive refinements of the last century, is a fact which nothing but ample experience could have established.

The advantage of the female sex *in the aggregate* is found to be four per cent. in longevity,—a difference sufficiently explainable by their comparative exemption from hard labour and bad weather; and this graduated diminution of advantage, as compared to that of the select classes, leads to a persuasion that no advantage whatever existed in favour of the uncivilized human female.

Physically as well as statistically speaking, an investigation of this question is of some importance; and the Parish Register Returns of the ages of the buried, may throw some light upon it. I ask your attention, therefore, to the fact, that although the females are double the number of males alive at 100 years of age (129 to 60 in the year 1821), yet the deaths of the females during the next ten years of life are in such proportion, that at the age of 111 the sexes are equal in number, viz. nine of each sex exceeded that age in eighteen years (1813-1830); and beyond that age the females died earlier than the males; so that the two last survivors were a female, who died at 120, and a male, who died at 124 years of age."

Mr. Rickman presents in the Gazette several tables illustrative of the increase and decrease of mortality, at particular years, at decennary periods, and in the two sexes. But as the laborious nature of the calculations is heightened by the obscurity of Mr. Rickman's style, not one medical reader out of six would be otherwise than mortally puzzled, at hunting the meaning of the zealous and amiable dealer in deaths. Leaving many tables and some most formidable diagrams to actuaries, we must content ourselves with picking out a fact or a conclusion here and there, for our edification or amusement.

### 3. *Increased Value of Life in this Country.*

The increase of the value of life in this country has been remarkable. We are the most long-lived (so far as calculations can enable us to ascertain any fact of the kind) of any modern or ancient people. Nay the whole mass of the people now live much longer than its higher classes did in the seventeenth and eighteenth centuries.

"The period," says Mr. Rickman, "of remarkable increase of the duration of life in the mass of the population is evident enough in the *stationary number of burials* during thirty-five years (1780—1815), while marriages increased from 64,000 to 89,000 (39 per cent.), and the population in larger proportion (about 50 per cent.), from the combined effect of increasing longevity, and the usual excess of births as compared to deaths in a thriving community. I do not know that the prolongation of life can arise from any proximate cause other than the increase of bodily health; nor that, from any thing more recondite than increasing plenty and comfort pervading all classes of society."

It is impossible to avoid agreeing with Mr. Rickman in this sentiment. But he indulges in another not quite so unexceptionable. He hints that the war and the war's expenditure were excellent things, and that they have produced an astonishing concentration of national prosperity in our fortunate island. Even Tories will exclaim that this is a little *de trop*, and be-taxed John Bull will growl that it is too bad on the part of the statistician. Of course we do'nt intend to take up this horrible heresy in politics, but we cannot avoid pointing out a circumstance, which appears to militate against our author's notion of the manifold advantages of war. Increased health and longevity in England would seem to have attained their maximum about the year 1818, and have since that period been rather on the decline. Now all contend that whilst the war lasted all went well enough. Then there were high prices and profuse expenditure. But when the war ceased, and the artificial impulse to trade subsided, then unfortunately the bills were to be paid, and then John Bull found out his blunder. If statistics go for any thing in such a matter, they seem to shew that bill and tax paying is fatal work.

### 4. *Mortality of Doctors.*

We are naturally deeply interested in the question—are we longer or shorter-lived than other classes of the community? There is necessarily much difficulty in ascertaining this point. No distinct records of the births and deaths of doctors are extant; and those who arrive at conclusions on the subject must take for data the lives of physicians and surgeons of eminence. But such data are very likely to prove erroneous. For, in our profession, those who attain eminence are probably longer-lived than the bulk of their brethren.

However this may be, Professor Casper, of Berlin affirms that the medical practitioners of Germany are shorter-lived than the members of other professions. M. Du Bois, has arrived at the opposite conclusion. Of 850 medical practitioners, he ascertained that 7 died between 20 and 30 years of age; 57 between 30 and 40; 83 between 40 and 50; 136 between 50 and 60; 202 between 60 and 70; 213 between 70 and 80; 116 between 80 and 90; 31 between 90 and 100; and 4 between 100 and 106. In fact, 365 of the 850 attained to 70 and upwards, which even exceeds the proportion allotted to the long-lived theologians, of whom, according to Casper, 42 per cent. attain the age of 70." The materiel of these statements is taken from Eloy's Dictionnaire Historique.

The anxieties of the medical practitioner, his midnight watchings, and the contaminated atmosphere he often breathes, must be sources of disease and of mortality. But then, on the other hand, he has one great source of safety open to him, which must tend to render the balance between him and his patients even—he takes monstrous little physic. Whatever may be the real state of the case, one practical rule is quite clear—we must all try to live as long as we can, were it only for the honour of the profession.

### 5. *Comparative Mortality of the Married and Unmarried.*

Dr. Casper of Berlin, himself, we suppose, a married man, has published an Essay to prove that the married are much longer-lived than the single are. He asserts that, in the case of females, the mean duration of life for the married woman of 25, is above 36 years ; while for the unmarried it is 30½. At 30, there is a difference of four years in favour of the married ; at 35, two years, and so on. With respect to men, he asserts from Deparcieux's and the Amsterdam tables, that the mortality of those from 30 to 45 years of age is 27 per cent. for the unmarried, while it is but 18 for the married ; and that for 41 batchelors who attain the age of 40, there are 78 married men. The difference becomes still more striking as age advances : at the age of 60, there are but 22 unmarried men alive for 48 married ; at 70, eleven bachelors for twenty-seven married men ; and at 80, for the three bachelors who may chance to be alive, there are nine benedicts. The same proportion very nearly holds good with respect to the female sex ; seventy-two married women, for example, attain the age of 45, while only fifty-two unmarried reach the same term of life. M. Casper, in conclusion, considers the point as now incontestibly settled, that, in both sexes, marriage is conducive to longevity.

Mr. Rickman has very properly and ably exposed this libel on batchelors and maids. He has shewn, satisfactorily that no material difference is appreciable in their respective longevities. The whole of M. Casper's conclusions are based in miscalculation and error. So let our younger readers take heart. At the same time, practical experience convinces us that, in the female sex, many diseases result from the inability to obey the dictates of nature. Who does not every day witness the thousand forms of hysteria with its many consequences, in unmarried women. And can any one believe that this is not prejudicial to longevity and health. The statisticians may, from circumstances, be unable to determine the numerical differences in the deaths of married and unmarried females, but the surgeon and physician who see human nature in detail, cannot shut their eyes to the obvious fact—that whether the unmarried can be proved to be as long-lived as the married or not, they are certainly subject to more miserable maladies, and their health is impaired, if their existence is not shortened.

With males the question is widely different. Fortunately or unfortunately for themselves, they are not restrained from sexual gratifications, like the weaker sex. Marriage then, in their case, cannot be sexually very important, and its influence must be exerted on their general habits and feelings.

### 6. *Statistics of Factories.*

Mr. Rickman assures us that he never could discover " any fact which was likely to place the health of the manufacturing population below that of other occupations." The expression, like the opinion, is loose. If Mr. Rickman means to say that, cæteris paribus, confinement for a great many hours in the day to the interior of a factory—exposure (as in many instances there is) to a very heated, a foul, or a dusty atmosphere—and a residence in the crowded lanes of a city, are circumstances no more likely to impair health and curtail life, than the avocations of the husbandman, or even the mechanic trades not exercised in the manner we have mentioned, we can only say that we marvel at what the science of statistics tends to. Mr. Rickman justly observes that longevity is only a term for health. We presume that the usual signs of the latter are recognizable by the medical man. Who, then, that sees the white-faced lean mechanic and the ruddy and robust ploughman, can doubt on which side *health* inclines. We are far from joining in the tumultuary cry against factory labour, but we cannot shut our eyes to the fact, that the great mass of the medical evidence pointed to its general unhealthiness. Some thought this greater, some less; but all, we believe, who have examined the question, have agreed that such labour is in the main unhealthy. It would be paradoxical in the extreme to assert that what injured the health would prolong the life. We recommend Mr. Rickman to look at the work of Mr. Thackrah, on the diseases of the manufacturing part of the people.

---

### Periostitis of the Orbit.*

Mr. Hamilton, an Irish surgeon, has related a case of periostitis of the orbit, which is practically, well worthy of attention. The particulars of the case are these.

*Case.* "Mary Falkner, æt. 33, florid complexion, married, and has had three children; the two first alive and healthy, the last a miscarriage, at seven months, came to me at the South Eastern Dispensary, complaining of great pain in the left eye, and side of the head, with impaired vision. A considerable protrusion of the eye-ball was at once observable; the eyelids, especially the upper, were swollen and puffy looking, filling up the usual depression beneath the eye-brow, of a dull red colour, streaked with veins. The eye-ball presented no marks of inflammation, a few tortuous veins being alone seen at its upper and inner part. The iris was of a greenish hue; the pupil natural as to size, but not shape, being oval from side to side, and at the upper and back part a bright green spot, of irregular shape and metallic lustre, was very distinct. The pain complained of was most intense, referred to the eye-ball, but darting into the head, the whole left side of which was affected; worse at night, and depriving her of sleep. It was aggravated by the least motion of the body or eye, and by lying on that side. There is also a distressing feeling of sand in the eye, probably produced by the friction of the tense eye-lids over the protruded ball. Sight is much impaired, objects appearing misty and indistinct, and on looking down double, muscæ volitantes are constantly before the eye. She is subject to giddiness and lightness of the head; pulse quick and full; tongue furred.

Three months ago she miscarried, and supposes she got cold; the eye became painful, and the eyelids red and swollen. Since then the pain has gradually increased, and vision equally diminished."

Mr. Hamilton was inclined, from the metallic lustre at the bottom of the eye, to consider the case one of incipient fungus hæmatodes. But the apparent health of the patient, and the continuance of vision, led him to doubt the correctness of this opinion. Not being able to detect the presence either of an abscess or a tumor in the orbit, he treated the case as one of an inflammatory nature, by leeches, cupping, blisters, and purgatives. But as the symptoms grew worse, he sent the woman into the Meath Hospital. Her state at that time is thus described.

"The prominence of the eye was now so much increased, that it had the appearance of being larger than the other, and the eyelids could not be completely closed; the protrusion was in the direction downwards and outwards. The pupil, dilated a few days before by the extract of Belladonna, has never since returned to its natural size, but now appears prevented from closing by the lens being pushed against it; it had still the same oval form; the lower edge of the iris was turned in; the metallic spot was much more forward, and now seemed to occupy the whole of the pupil, giving it a greenish and rather opaque look; a small, brown, waving line, like a vessel, was seen crossing it. She got no relief from the pain night or day, and though mitigated for a short time, by leeching, it

* Dublin Journal. No. XXVI.

soon returned worse than ever; the stomach, too, became so irritable that nothing would stay on it, and the vision was reduced to distinguishing dark objects between her and the light. Though the irritability of stomach was allayed by the application of a blister, the other symptoms became worse, in spite of a variety of treatment, and she left the hospital hopeless of getting any relief."

The general impression on the minds of the surgeons of the hospital, was that of the disease being malignant; and, but for the deep-seated pain in the head, the eye would probably have been extirpated.

About a week after the dismissal of the patient from the hospital, Mr. Hamilton discovered that, on pressing hard on the orbit, its whole upper and inner part was extremely tender. It struck him that the disease was periostitis. On questioning the woman closely, she admitted that she had been disordered by her husband eight years previously, and had taken mercury; that shortly afterwards an eruption, with what seemed to have been iritis, shewed itself; and that sore throat followed, with occasional pains in the bones for the last few years.

Mr. Hamilton now put her on calomel and opium, with the decoction of sarsaparilla. At the end of six weeks' salivation, she had lost all pain, and regained her health and spirits; the eye had nearly returned into its proper place in the orbit, the swelling had disappeared from the lids, and vision was sensibly improved. Some months afterwards, when Mr. Hamilton met her, there was no difference in the appearance of the eyes, the sight, though still misty, was otherwise tolerably good, and no return of the pain had taken place.

Mr. Hamilton indulges in many remarks, which do not seem to require any notice. The case itself tells its own story, not an uninstructive one. We cannot forbear expressing some surprise, that a suspicion of the nature of the case, or at all events a trial of the appropriate remedies should have happened at so late a period. Tenderness on pressure is a physical sign, readily appreciable by a manual examination, whilst the unnatural protrusion of the eye, would naturally lead to such an investigation. Independently, however, of any such physical evidence of inflammation of the bone or periosteum, the mere existence of severe deep-seated pain, connected apparently with some inflammatory affection of the tissues, would induce the majority of practical surgeons or physicians to prescribe calomel and opium. The symptom is one, to which, as a symptom, that remedial combination is peculiarly adapted. The particulars of the case are calculated to give an useful hint to its readers.

---

### On the Use of the Cochlea in the Organ of Hearing.

We need scarcely observe that the functions of the various parts that compose the internal ear in man, are imperfectly understood. That so complicated an apparatus should be essential to the perfect sense, is consistent with analogy and reason. But physical science has not hitherto informed us, what part is played by each component section of the mechanism, nor what is the individual office of the cochlea, the semicircular canals, and so forth.

Weber, a physiologist of no mean rank, has laboured to prove that the office of the cochlea, is to appreciate those sounds which are transmitted through the cranium as a solid. The opinions and the reasoning of Weber on this subject are presented to the English public by Dr. Graves, the able Editor of our Dublin contemporary. We shall introduce such portions of his paper in that Journal, as will put our readers in possession of the main points which are urged in favour of the hypothesis in question.

"It is evident," says Weber, "that the propagation of sound to the internal ear, takes place not merely through the *meatus auditorius externus*, but also through the *bones of the cranium*; by the former we receive notice of sounds from without, by the latter, we more readily hear our own voice. The vibrations produced by our own voice are indeed also heard by the route of the external ear, but they are conveyed with greater distinctness through the medium of the bones of the skull. Thus, if you stop both ears firmly with the fingers, so far is your voice from being rendered inaudible, that you hear it more distinctly and louder than before. If now we remove the finger from one ear, immediately we find that the sound of our voice appears stronger in the other. *I shall now endeavour to prove that sounds propagated by and conveyed through the bones of the head, are heard chiefly by means of the cochlea, whereas sounds coming from without by way of the meatus auditorius externus, are not so readily received by the cochlea,* as by the route of the vestibule and the semicircular canals."

Weber observes that in almost all animals, the vibrations on which sound depends are communicated to the extremities of the acoustic nerve, by the two-fold means of a vibrating solid and a vibrating fluid. Why it should be so, is not at present clearly understood, but M. Weber asserts that it is the fact with regard to the greater number of animals.

Sounds lose comparatively little of their force by propagation through an uniform medium; but the contrary is the case when they pass from one medium to another, as from a solid to a fluid, or from a fluid to a solid. Thus sounds transmitted through water may be heard at great distances, while the head is under water; but they become inaudible the instant that the head emerges above its surface.

" Now it is found," continues Weber, " that solid bodies communicate their vibrations to fluids, with a facility proportioned to their extent of surface, and that solids receive vibrations from aeriform media more easily when the solid is shaped in the form of a membrane. A tense cord does not easily communicate its vibrations to the air unless it be fixed to some flat body, which, being of a like nature solid, receives the vibrations of the string without difficulty or loss, and propagates them to the air through the medium of its own extensive surface. It is an observation of these phenomena which has led to the adoption of sounding boards in those musical instruments, when the vibrations causing sound proceed from strings, as in case of the violin, the piano, and the harp, whereas sounding boards are not required in the various species of wind instruments. The reason of this difference is sufficiently obvious, in the one case a solid with an extensive surface must be brought into connexion with the vibrating string in order to diffuse its vibrations more energetically through the air, whereas in wind instruments, the vibrations being derived from the air itself, no such provision is necessary."

The two preceding facts may be considered as the premises of Weber's argument. The conclusions follow thus.

The sonorous vibrations of the air in the mouth pass to the internal ear through the medium of the cranial bones. They impinge by preference on that portion of the acoustic nerve which is distributed upon the cochlea, *because* on it the nervous expansion is in intimate relation with its walls, which are themselves connected with the cranial bones. But the acoustic nerve where distributed through the vestibule and semi-circular canals is not so favourably circumstanced for receiving the vibration of the cranial bones, for here the nervous expansion is separated from the bony parietes either by means of a liquid secretion or of a loose cellular membrane. In accordance then with the law that sound is transmitted with most facility when the medium is not changed, M. Weber supposes that the acoustic nerve in close connexion with the bony cochlea, is more readily affected by vibrations of the cranial bones: than in the vestibule or semicircular canals, where another medium separates it from the bone.

With the following remarks, which we cannot materially abridge, we conclude.

" The preceding observations," concludes Weber, " render it sufficiently apparent, that the membranaceous vestibule and membranaceous semicircular canals, differ in structure from the osseous vestibule and the osseous semicircular canals, in such a manner, that vibrations travel through the bones of the cranium with more facility to the cochlea and round sac, than they do to the membranaceous portions of the internal ear.

The next question is, whether the sonorous vibrations that are derived from the external air and proceed through the meatus externus to the ear, are propagated with greater facility and strength to the nerve of the vestibule than to the nerve of the cochlea. In the first place, it is evident that the vestibule and the semicircular canals directly connected with it, must receive a stronger impulse from the aerial vibrations than the cochlea, for the former have a solid communication by means of the chain of ossicula with the membrana tympani, whose vibrations are consequently imparted at once to the membrane of the fenestra ovalis, whereas no such direct communication exists between the membrana tympani, and the fenestra rotunda. The membranes of the semicircular canals and the vestibule too, seem to be more easily set in motion by the fluid which invests and surrounds them, than is the case with the lamina spiralis of the cochlea. The latter is, nevertheless, furnished with provisions calculated to enable it to receive impulses from the external air also; for the fenestra rotunda, being closed by a membrane, must impart the vibrations that occur in the cavity of the tympanum, while another opening, by forming a communication with the vestibule itself, must place the cochlea in connexion with the latter, in such a manner that the vibrations which the chain of ossicula have imparted to the fluid of the vestibule, must through that fluid be at once propagated to the cochlea.

I have next to prove the assertion which I made at the commencement, viz. that nature has so constructed the ear in man, and various other animals, as to make a provision for the reception of the sonorous vibrations in a two-fold manner, by means of the acoustic nerve, which is so disposed in the internal ear as to present, for receiving these vibrations, a double surface of contact; the one consisting of a soft, pultaceous, nervous expansion, surrounded by a fluid, the other formed of a reticulated net-work of extremely minute, but firm, nervous, ramifications. The former receive the sonorous vibrations through the medium of a fluid, the latter of a solid. In man the cochlea is the portion of the organ where the firmer extremities of the acoustic nerve are disposed for this purpose. Fishes and amphibious animals have no cochlea, but they have an arrangement which answers the same purpose; for in osseous fishes, we find that the membranaceous labyrinth contains three white little stony bodies, of great specific gravity, very hard, and much resembling vitrified argillaceous clay; two of these are enclosed with a sac full of fluid, adjoining the vestibule, and lodged in the basilar portion of the occipital bone; these lapilli are furnished with fine nervous filaments, fastened to rough depressions and elevations on their surface; thus vibrations are imparted from the lapilli to the acoustic nerves; the third lapillus is situated in the anterior portion of the membranaceous vestibule, and receives no nervous filaments. It has, however, another mode of communicating with the acoustic nerve, for it lies against a very large branch of that nerve where it expands on the membrane of the vestibule, and thus compresses this nerve between itself and the cranium. In cartilaginous fishes and in amphibious animals, in the place of these lapilli, we find certain little bodies consisting either of concrete gelatine or of a chalky pultaceous matter, and to which both Scarpa and myself have traced ramifications of the acoustic nerve; neither is the *lamina spiralis* of the cochlea in man formed without reason of two structures, an osseous, and a cartilagino-coriaceous tissue; for as the same nervous branches pass from the osseous to the cartilaginous portion of the *lamina spiralis*, it is natural to conclude that receiving sonorous impulses from both, these impulses are communicated by means of a different mechanism in the two cases. This idea is confirmed by an examination of the calcareous fragments found in the labyrinth of Rays, and which are composed of two portions; one pellucid and resembling a tremulous jelly, the other white and chalky, seem to discharge the same function in these animals, that the cochlea does in man. These fragments are so divided, that their gelatinous and chalky portions lie in contact by means of extensive smooth surfaces. Nature has so disposed the extremities of the auditory nerves in the semicircular canals of all animals, that these extremities receive the sonorous impulses directly from a fluid. This is very plain, even in man, where the dilatations termed ampullæ, and which correspond with a similar enlargement of the nerve, are both filled with and surrounded by a fluid. In fishes it is still more evident, for in Rays, a nervous filament can be traced to each ampulla, which it enters and forms within a crescent-like septum."

We fear that ingenious as the preceding reasoning must be owned to be, it is still extremely speculative. When we reflect on the facility with which sonorous vibrations are communicated through the whole body, composed as it is of media differing much more in nature and extent, than the nervous expansions on the cochlea and the semicircular canals can do, we are almost involuntarily led to pause before we yield implicit confidence to M. Weber's conclusions. Unfortunately, in the case of the internal ear, decisive experiments cannot be performed, and the observation of pathological changes in connexion with alterations of the functions, is attended with nearly equal difficulty. The amount of confidence extended to M. Weber's opinions will vary with the turn of mind of the individual who peruses them. One man yields assent to the evidence of probabilities, with much less difficulty than another. Whatever may be the actual truth of M. Weber's supposition, it is certain that it is ingenious, and possessed of plausibility. We think Dr. Graves entitled to many thanks, for bringing this as well as other Continental papers before the profession in this country. This is not the first time, nor, we are sure, will it be the last, that we have had or shall have occasion to express our sense of the zeal and the talents of that gentleman.

---

### PROFESSOR HARRISON'S ACCOUNT OF CRYSTALS OBSERVED ON THE PERITONEUM OF THE HUMAN SUBJECT.

Mr. or, rather, Dr. Harrison, who is well known as the author of an excellent work on the anatomy of the arteries of the human body, has communicated to our Dublin contempory*—An account of some crystals, apparently of the ammonio-magnesian phosphate, which he has observed on several occasions on the peritoneum of the human subject. He is not aware, nor are we, of any published account of these crystals prior to his own.

" It is now," he says, " some time since my attention was first accidentally directed to these, by the sensation which they gave to my hand, when removing some of the viscera of the abdomen, in the course of general dissection; I have since met with several examples, in all I should think about five or six, and in these the greatest similarity prevailed. The crystals, though extremely small, are very distinct, they are prismatic and exhibit clear and brilliant facets; before removal from the membrane they appear semi-transparent, not very unlike the colour of the peritonæum itself, hence they are usually detected by the touch rather than by the sight. They are principally to be found in the lower regions of the abdomen: in the first case I met they were in great abundance on the cæcal and iliac portions of the peritonæum; I have since found them in both iliac regions, as also in the inguinal fossæ, also along the course of the colon and the forepart of the rectum, but not on the bladder; I have seen a few on the mesentery, and on the terminating convolutions of the ileum intestine; but in all the examples I

* No. 26.

have met, they were most abundant in the iliac regions. I have not observed any on the stomach, liver, spleen, or duodenum, or on any part of the peritoneum in the upper regions of the abdomen; in the lower part of the cavity they were not confined to the visceral, though certainly they were more abundant on it than on the parietal portion of the membrane."

Dr. Harrison cannot consider these crystals as actually a morbid product, nor has he found them in connexion with diseased structure. They have almost always occurred in the bodies of females, emaciated, and advanced in years. As the crystals occur where no liquid effusion is present, they cannot be considered a mere crystalline deposition from it. The crystals, too, were not loose and unattached, so as to admit of being rubbed off, but, on the contrary, they were set very close together, and were intimately connected to the membrane by a very fine but still a resisting film, or a sort of albuminous pellicle, which was continued from the peritonæum over the base of the crystal, on which it was imperceptibly lost. In order to detach any of them it was necessary to scrape them off with the knife; on their removal, however, no orifices were observable in the membrane, and the latter appeared in a state of integrity, and free from any abrasion. When these crystals were removed and washed, they became perfectly transparent, their prismatic form and polished surfaces were then also very apparent.

Dr. Harrison collected some, and sent them to Dr. Apjohn for analysis. He found that they were composed of phosphoric acid, ammonia, and magnesia. What the causes of their deposition may be, or what are even the attendant circumstances, we are unable to determine. Dr. Harrison has never seen them in the pleuræ or the pericardium.

"I once, and only once, met with a very slight trace of the same formation on that portion of the arachnoid membrane which covers in a very loose manner the cerebral protuberance or pons Varolii, but the crystals were not in sufficient quantity to enable me to institute an accurate comparison. Finally, I may add that, a short time since, when examining the head of a child about seven years of age who had died of hydrocephalus, and in which the arachnoid membrane on the inferior surface of the medulla oblongata was inflamed and thickened from tubercular deposit, I detected a small patch of the membrane to be in a very rough condition; this, on close examination, I perceived was partly owing to the presence of very distinct small short crystals of a brownish colour and which were intimately adherent to this diseased and thickened membrane; the quantity of these, however, was so very small, and the parts were so much disturbed and injured by the examination, that I was not able to institute any more accurate investigation as to their nature and properties; from their general appearance, however, as well as from all the concomitant circumstances, I should consider these as a totally different product from that which I have first described as found in the peritonæum."

---

### Maltreatment of a Fractured Leg in Lafayette. By Boyer and Deschamp.

The following anecdote of execrably bad surgery on the part of Boyer and Deschamp, and of most surprising patience on the part of General Lafayette, is told by Jules Cloquet, and extracted from his work by our Dublin contemporary.

"During his last illness, Lafayette related to us more than once the particulars of the treatment he underwent in 1803, for the fracture of the thigh, caused by falling from a height; Deschamp and Boyer, whose memories I respect, and under whose care I was educated, was called in, and enclosed the limb in a machine constructed so as to maintain it in a state of continual extension. Lafayette having promised his surgical friends, that he would patiently endure the pain and inconvenience necessarily attendant on the treatment, did not give utterance to a single complaint, and did not betray the least sign of being in pain for the whole of the twenty days which elapsed before the apparatus was for the first time removed; when it was removed, however, his surgical friends could not conceal their consternation; Deschamps turned pale, and Boyer appeared thunderstruck. The bandages of the upper portion of the apparatus had, by their pressure, cut deeply into the flesh, and exposed the femoral artery, while the skin of the dorsum of the foot was rendered gangrenous by the inferior bandages, so as to lay bare the extensor tendons of the toes. More than five weeks were required to heal these wounds, and when the cure was completed, an almost complete anchylosis of the hip joint had taken place, so that he was lamed for life."

Well might Deschamp turn pale, and Boyer be thunderstruck, for their's was no mean carelessness. This is just the case for Mr. Radley. It would prove to demonstration the mischiefs of the practice of extension. Fortunately, *such* extension is not common.

---

EXCISION OF THE CYST OF A STRANGULATED UMBILICAL HERNIA. SUPPOSED RADICAL CURE.

The following case is contained in our valuable transatlantic contemporary—the American Journal of the Medical Sciences.* The reporter of the case is Dr. Heustis, of Mobile, who performed the operation.

*Case.* On the night of the 7th of June, 1829, Dr. H. was summoned to a female slave. The woman was apparently dying; in extreme pain, with an abdomen immensely swollen; pulse feeble and intermitting; body cold, and covered with a copious exudation of clammy sweat. The protrusion of the umbilicus was about the size of one's fist, and had been increasing for two days. A physician had been called in at an early period, who had in vain attempted the reduction by the use of all the ordinary means, such as bleeding, warm bath, taxis, &c. He had, however, left her, with the intention of returning in the morning prepared for the operation. But in the meantime the patient had continued to grow worse. The abdomen was tense, and very painful to the touch, there was frequent vomiting, and there had been no motion from the bowels since the commencement of the symptoms of strangulation.

"Finding that there was no time for delay, I proceeded to the operation by candle-light. The integuments were carefully dissected from the distended sac, and the point of protrusion arrived at, almost entirely obliterated, and not sufficiently large to admit the little finger. The sac was now divided, and the opening enlarged with the blunt-pointed bistoury, so as to admit the protruded portion of the bowel, which was dark-coloured and almost gangrenous. There was still, however, a considerable volume of hernial sac, consisting of the protruded and distended peritoneum, much thickened from former and repeated attacks of hernia. To leave it would be exposing the woman to future returns of her habitual infirmity; whereas, by removing it, a radical cure would, in all probability, be effected. I accordingly removed it entirely with the scalpel, close to the point of protrusion. No sooner were the dressings applied than a free discharge from the bowels took place, to the great relief of the patient.

The cold perspiration had now disappeared; the pulse had acquired more firmness, and the tension, pain, and hardness of the abdomen diminished. In the morning the abdominal distention had subsided, and the woman was in a fair way of doing well. I left her, giving the necessary directions for her future treatment, and saw her no more. Some time subsequently, I learnt from her master, that her recovery was speedy, and that she had no more returns of the complaint, a radical cure having been effected by the operation."

The preceding description might be clearer. It is certain that the hernial sac was removed, and that no bad con-

* No. XXXII. August, 1835.

sequences followed. But it is not certain that a radical cure was effected, nor that the practice would, in similar circumstances, be advantageously, or even safely imitated.

---

On the Sounds of Substernal Aneurysms. By Dr. Henderson.*

Dr. Henderson makes an observation, which now hardly needs to be supported: that although an exact diagnosis of intractable diseases may neither directly contribute to their alleviation or their cure, still it is useful to the physiologist and the physician. The fact is, that a precise knowledge of the symptoms and signs of any one incurable malady, presupposes, in some degree, a precise acquaintance with the symptoms and signs of other diseases, which may or may not be susceptible of relief. If we recognize a certain malady present, we are enabled to ascertain its absence, for the knowledge of the characters of one disease is, pro tanto, the power not only of distinguishing it from others, but also of distinguishing others from it. They who exclaim against exact diagnosis are usually shallow persons, who are deficient either in the ability or the industry required for the successful analysis of symptoms. But to resume.

The nature of Dr. Henderson's communication is briefly and satisfactorily stated by himself. His remarks have been derived chiefly from cases which were of a sufficiently obvious nature, and which were studied with the purpose of acquiring means to detect the more obscure instances of the disease by a consideration of the anatomy and signs of the more apparent. In order that our readers may understand the signs of substernal aneurysms, it is necessary that they should become acquainted with the varieties in those aneurysms, to which Dr. Henderson attaches some importance.

"The signs," he says, "of substernal aneurism are subject to variety from the influence of form, position, and the presence or absence of *coagula.* That part of the *aorta* which includes the arch and the ascending portion of the vessel is liable to be affected differently from the other arteries of the body, by acquiring a large size from nearly uniform dilatation, and is also subject, like them, to the sacculated form, resulting from more or less rupture of the inner and middle coats. The external coat and the membranous substances that invest it may be distended to a very large size, while the communication betwixt the cavity of the aneurism and the *aorta* may not equal half an inch in diameter. It is, indeed, remarkable through what a small aperture a fluid power may be directed capable of causing enormous distention of the outer coat. Scarpa quotes from Palletta a case of aneurism of the thoracic *aorta* which had attained a great size, while the opening that led from the vessel was incapable of admitting a substance larger than a pea. The communication is, however, in the majority of cases, much larger, so large, indeed, in some, that the sac may have a diameter at its mouth equal to that at any other point of its extent. The relative size of this opening seems to have a material influence on the sounds emitted by the aneurism.

Aneurismal sacs may, or may not, contain *coagula.* When present, these may be arranged in concentric laminæ within the aneurism, so as to present no obstacle to the free passage of the blood through the contiguous part of the vessel, or they may project into the vessel, and oppose themselves to its current, a remarkable instance of which is afforded by Case I. Sacculated aneurisms of considerable size are not commonly altogether void of *coagula.* One of this kind I had an opportunity of witnessing, in which the sac was as large as an infant's head, and the opening leading into it but two inches in diameter; yet it contained no deposition of fibrine. It had produced death by the discharge of its fluid contents through a small slit into the cavity of the right *pleura.*"

---

* Edinb. Med. and Surg. Journal, No. 127.

Although the ascending aorta may yield in any part of its circumference, yet the direction of the aneurysm is generally towards the right side.

A substernal aneurysm may give rise to two successive sounds, corresponding in point of time to those of the heart, or may occasion but one sound, synchronous with that of the ventricles. These are to be understood as originating in the aneurysm, and require for their production that it should be altogether or nearly without *coagula.* The sounds which are heard over the aneurysm, when filled with coagulated fibrine, are different both in their nature and in their origin; they are transmitted by, but do not originate in, the aneurysm. Dr. Henderson is disposed to believe, from his own experience, that when two sounds originate in the aneurysm, they always possess more or less of the rasping noise, but when one only has its rise in the tumor, it may have either this rasping sound, or may be pure, and, in some measure, similar to the ventricular sound in health. Dr. Henderson enters into many considerations, with respect to what the sounds might or might not depend on ; but, as none but profound auscultators can follow him, and as they are too much founded on supposition to be satisfactory, we must refer our readers to our contemporary for their perusal. The following observations on the signs of enlargement of the calibre of the arch of the aorta, will probably be more generally understood.

" Of the sounds accompanying general enlargement of the calibre of the ascending *aorta* and arch, little need be added to what has already been said of sacculated aneurisms with a very wide passage leading from the *aorta.* When complicated, as it sometimes is, with disease of the aortic valves, and permanent patency of the aperture surrounded by them, this kind of aneurism may present two rasping sounds along the *sternum.* In order to distinguish this state of disease from the sacculated aneurism, at the orifice of which two rasping sounds are produced peculiar to itself, it will suffice to remember what has already been said regarding the second sound in cases of permanent patency, and that in mere dilatation, the impulse is much less on any part of the *sternum* than immediately above this bone.

The presence of a hoarse rasping sound in the carotids is characteristic of the several forms of substernal aneurism which have not been consolidated by *coagula.* It is likewise occasionally met with in these vessels in cases of considerable narrowing at the mouth of the *aorta,* especially when complicated with greatly increased power of the heart. The state of percussion and the absence of preternatural impulse in the course of the *aorta* will enable these cases to be distinguished. The pathological conditions necessary for the production of these sounds in the vessels beyond the aneurism are merely the existence of a considerable expansion beyond a part of much more limited capacity,—conditions which are characteristic both of aneurisms and of the valvular disease referred to. Roughness of the inner surface of the aneurismal dilatation, or of any other part of the apparatus, does not seem to be requisite."

Aneurysms contiguous to the heart, when filled with fibrine, become mere passive instruments for the transmission of the two sounds of the heart itself. Why they should do so must be evident to every one. Cases of this kind cannot be confounded with that very different form of aneurysm in which the sac is still capable of admitting blood at each contraction of the ventricles. In the latter, it is true, there are two sounds, both of which may be free from murmur, but the first is louder than that of the heart, and most likely results, as has been said, from the shock of the sac or its fluid against the *sternum* or ribs, while the second can be accounted for only on the supposition that it is transmitted from the valves. Besides, aneurysms not filled with *coagula* have a much stronger impulse than the others. Yet, in aneurysms *not* consolidated, the second sound is liable to many variations, some of which are not readily accounted for.

On the whole, Dr. Henderson arrives at the following conclusions, which we subjoin. Our readers may test them in their bed-side experiments.

"1st, That aneurisms of the ascending *aorta* may or may not have sounds originating from themselves.

2d, That when two sounds *originate* in the aneurism they are produced at the opening by which it communicates with the *aorta*, and that only sacculated aneurisms can originate two sounds, and these of a more or less rough character.

3d, That when two pure sounds, *i. e.* sounds free from morbid murmurs or rasping, are heard in the situation of aneurism, one only originates at the tumour, the other being communicated from the sigmoid valves.

4th, That the second sound of the heart is susceptible of increase, and probably of diminution, from diseases of the *aorta* capable of modifying the energy with which the blood recoils upon the sigmoid valves.

5th, That aneurisms near the heart, consolidated by the formation of fibrinous concretions in their interior, transmit the sounds of the heart, and that these sounds diminish on being thus transmitted.

6th, That it is immaterial in the diagnosis of substernal aneurisms, whether the first sound be rough or pure.—See Dublin Journal, vol. V.

7th, That it is equally immaterial in the diagnosis of these aneurisms, whether the second sound be rough or pure; that, if the former, it can be the result only of one of the two following conditions, either of a sacculated aneurism *originating* two sounds, or of permanent patency of the aortic opening.—See Edinburgh Medical and Surgical Journal, No. CXXII.

8th, That it is immaterial in the diagnosis of substernal aneurisms, whether the second sound (if pure) be heard more or less distinctly in the region of the heart, than nearer the seat of the aneurism.—See Bertin, p. 95: Hope, Lond. Med. Gaz. 1829, &c.

9th, That, although certain preternatural sounds at the upper part of the *sternum*, or on the superior part of either side of the chest in front, together with dulness on percussion and morbid impulse at the same places, afford means of detecting the great majority of substernal aneurisms, yet it is possible for an aneurism consolidated by *coagula*, and without external tumour, to exist without our being able to distinguish it from other solid masses, alike capable of conducting the two sounds of the heart, and communicating its impulse."

---

### Case of Recovery from Dislocation and Fracture of the Spine.

Dr. Dorrance, of Amherst, Massachusetts, relates a case of this sort, which possesses some features of practical interest.

*Case.* About six years ago Amos Marsh, of Sunderland, while working in the woods, was struck by a falling tree, and bent to the ground. Dr. Dorrance saw him soon after his removal to his house. He found paralysis of the lower extremities, and a projection at the eleventh dorsal vertebra, where an angle of forty-five degrees was formed.

"It looked like so easy a thing to make the spine straight, that I could hardly resist the inclination to put it so. And the by-standers were impatient at my hesitation to do it. I supposed there was partial dislocation of the vertebræ, which any attempt at reduction would probably make a perfect one. I knew, too, that dislocation could not take place without fracture of the spinous or transverse process, and that loose spiculæ of bone would very possibly be driven into the spinal marrow, and cause instant death. A consulting physician, who saw the patient some hours after, was anxious to attempt a reduction; and when dissuaded from that, proposed cutting down and removing the broken and probably depressed fragments of bone. It was, however, concluded to trust the patient to nature, using bleeding and low diet to prevent, as far as possible, inflammatory action in the injured part.

Mr. Marsh had for a number of weeks almost perfect paralysis of the lower limbs and of the lower abdominal viscera. Urine was drawn off by the catheter, and the bowels moved by stimulating injections. By degrees, sensibility and mobility returned to the limbs, and the bladder and rectum resumed their functions. In four months the patient walked with crutches, and in six with a staff. In less than a year he resumed his trade, that of a cooper, and he now performs as much labour, sometimes in his shop and sometimes in the field, as most men of his age. There is a stooping of his back, and a sideway motion to his gait. The vertebræ are not in place, though more so than at first, and I believe much more so than art could have placed them."

The event proved, as reasoning might have anticipated, that Dr. Dorrance displayed his judgment in this treatment of the case. In doubtful circumstances, the rule of practice should almost always be—to avoid interference, when the probability of benefit is dubious, and the risk is great. Unsuccessful operations are, as we have often observed, prejudicial to the interests both of humanity and science. They create or confirm the too general doubts in the efficacy of the latter, and they prevent many persons from submitting to operations which would be really successful.

Strongly as the propriety of cutting down upon the vertebral column, and removing the depressed portions of bone, has been advocated by some able surgeons, facts and reasoning are so powerfully opposed to it, that the profession may be said to have decided against it. It is generally admitted now, that the analogy between the fractured spine and fractured cranium is too imperfect, to allow of arguments founded on the issue of treatment in the one case, to be applied with strict propriety to the other.

But occasionally patients exhibit a displacement of the vertebræ after injury, which appears to be remediable by moderate extension. Should such extension be resorted to? This is rather a difficult question, and the answer must depend on the judgment of the surgeon, and, indeed, on the concurrent circumstances in the individual case. We have seen one instance, and heard of another, in which extension seemed to be useful. The case which we saw was that of a boy, admitted into St. George's Hospital many years ago, with apparently fracture of the spine, and projection backwards at an angle, of one or two of the dorsal spinous processes, about the middle of the back. There was paralysis of the parts below the seat of injury. As there was not much pain on pressure, and the youth of the patient was favourable to the attempt, cautious extension of the spine was made. The projection manifestly diminished, and, although we believe that no immediate alteration with regard to the paralysis occurred, yet the patient ultimately did well, and left the hospital, after some months, with a certain degree of power over the inferior limbs. The case of which we heard was received, like the last, into St. George's Hospital. But, in this instance, we believe that the extension was made prior to the admission of the patient, and he also did not die. What the precise state of the paralysed parts was, we cannot now pretend to say. In both cases the extension did no harm, if it did no good; but our impression is, that it seemed to most of those who saw the cases to be really productive of some benefit.

There is an observation on the part of Dr. Dorrance, to which we would direct attention. He remarks that, in the case of his patient, he used bleeding and low diet to prevent, as far as possible, inflammatory action.

Now this is a precaution neither, we conceive, sufficiently insisted on nor practised. Surgeons are too apt to overlook the occurrence of inflammatory action about the membranes or the substance of the spinal marrow, after fracture of the vertebræ. The depressed condition of the patient, the paralysis, the disposition to the occurrence of sloughs of the integuments below the injury, all concur to prevent the surgeon from watching for inflammatory symptoms, or from meeting them, if they are present, with the same degree of earnestness he exhibits when the cranium has been fractured. Yet the cases of fracture of the spine that have done best within the limits of our own observation, have been those in which small bleedings, cuppings, calomel and opium, and remedies of that description, were employed. Of course, we do not recommend the indiscriminate use of antiphlogistic measures in *every* case of fracture of the spine. All we mean to say is, that surgeons, perhaps, disregard them too much, and that where circumstances justify their adoption, they are both reasonable and useful.

---

### Successful Amputation during the Progress of Traumatic Gangrene.

The occasional propriety of amputating a limb during the progress of traumatic gangrene, is too well established to require much further illustration or enforcement. But, although the operation may, under favourable circumstances, be resorted to, our readers must not suppose that, on the whole, it is a very successful one. We have seen it performed six or seven times, but never once with success. If we were to trust what some of the continental journalists say, we should be led to believe that the operation is a very fortunate one. But this is a little *de trop*. We are sorry to say that amputation for traumatic gangrene is sufficiently unsuccessful in the London Hospitals, to render it advisable to encourage surgeons, by the publication of any authentic instances of a favourable termination. For this reason, then, we notice the succeeding case.

*Case.* Mr. Eve, Professor of Surgery in the Medical College of Georgia, was summoned on the 1st of January, 1835, to visit a black man, then under the care of Dr. Olive, and Dr. R. Williams. The patient, a stout fellow, 40 years of age, had, a fortnight previously, received among other injuries, a double fracture of both bones of the right leg, one fracture near the ankle, the other just below the knee. The limb was placed in splints, and for a week or ten days it appeared to do well, but mortification then commenced, and progressively extended. On the 1st of January, when Mr. Eve was summoned, the condition of the patient was as follows :—Right foot and leg entirely sphacelated, knee of same side gangrenous, right thigh infiltrated with serum, and under or posterior surface much thickened from position, with *distinct crepitation* over the anterior surface, extending as high as Poupart's ligament. The pulse was small, wiry and feeble, beating about one hundred in the minute; tongue furred, whitish: courage good. Under these circumstances, amputation of the limb near the trochanters was decided on.

" After pressure was made upon the vessels, an incision in a longitudinal direction was made on the anterior face of the thigh, when oily serum and blood flowed out, from which we felt encouraged, and amputation was performed in the ordinary manner, as already described in a preceding number of your Journal. The patient being supported with laudanum, brandy and water, bore the operation much better than was expected. The stump presented a very unfavourable aspect, the skin of very unequal thickness, owing to the deposition and thickening from position, the muscles were very dark, the arteries and the cellular tissue, particularly along the course of the absorbents, blackish, and from the hollow of the divided thigh bone, the medullary substance protruded of a dark purple colour. On examining the parts removed, the knee-joint was found disorganized—matter extending in the cellular tissue above it; and upon removing the projecting medullary substance from the os femoris, which resembled that of the corresponding part of the stump just noticed, *pus* made its appearance."

In a month or six weeks the patient entirely recovered.

The case is put forward by its author not only as an instance of recovery from amputation performed during the progress of traumatic gangrene, but of success when the knife cuts through parts actually implicated.

---

### PROFESSOR MUELLER'S ACCOUNT OF THE ARTERIES THAT PRODUCE ERECTION OF THE PENIS.*

Professor Mueller, of Berlin, has lately discovered, or, at all events, described the arteries of the penis which produce the phenomenon of its erection. We will present the most essential portions of his statements on the subject.

We need not remind our readers that the penis is almost exclusively supplied from the internal pudic artery. This ultimately splits into two branches, the artery of the corpus cavernosum penis, and the dorsal artery of the penis. The former penetrates the corpus cavernosum at its root, and ramifies within it; the latter, after sending twigs to the fibrous envelope of the penis, its fascia, outer skin, and prepuce, terminates by ramifying in the glans.

"The distribution of the principal branch of the arteria penis was all that was hitherto known of this important vessel: it is in the more minute distribution of its branches through the corpus cavernosum penis that the secret of the cause of erection has remained concealed. It is not a lucky accident, but a systematical arrangement, and investigations proceeding from certain combinations, that have guided me, in this instance, to an important discovery. It is generally thought that the arteria profunda penis, which nourishes the substance of the penis, is at the same time capable of filling the cells of the corpus cavernos. with blood during erection also; that the nutritive blood, as well as that serving for the purpose of erection, proceeds from the fine twigs of the same vessel into the fine (capillary) veins; that it passes inwards from these into the sinuous veins, and again from these to the vena dorsalis penis; and that the state of erection distinguishes itself from the ordinary state of circulation in this organ, partly in the quantity of blood circulating in its vascular net-work, and partly in an impediment offered to the return of the blood through the veins of the penis. The older writers, on the other hand, considered the sinuous veins of the corpora cavernosa to be cells;—imagined that the nutritive blood supplied by the ordinary circulation did not at all reach these cells, or pass through them, but was carried off by appropriate veins; and that still, during the state of erection, the cells of the corpus cavernosum were filled with blood. Both views are, as we shall see, incorrect. The opinion of the old writers is proved to be unfounded, as well by investigations on the dead body as by the vivisections of beasts. In many experiments performed on the living horse, dog, ram, &c., I have observed, that upon making an incision in the corpora cavernosa, these bodies were not, in their unexcited state, much charged with blood, but that blood is contained in their sinuous veins, although certainly in less quantity than in the corpus cavernos. urethræ, which bleeds freely when cut across. Farther, in the majority of human subjects blood is found in the sinuous veins of the corpora cavernosa penis."

M. Mueller, it appears, had long thought that erection did not depend on the arrest of the flow of blood in the dorsal veins of the penis. He had also thought that different branches of the artery of the corpus cavernosum served for the purposes of nutrition and erection. It was "one of the happiest days of his life" when he found this, his conjecture, true, and "the wonderful difference" between the two sets of twigs alluded to is described by the Professor as follows.

I. "*The nourishing Twigs* (*Rami nutritii Arteriæ profundæ Penis.*)

When an injection of the arteries of the penis is made with size and vermilion, a considerable portion of the injected mass is always forced into the cavities of the corpora cavernosa, as well in the human organ as in that of

---

* Medical Gazette, Jan. 9, 1836.

the horse and dog. When this mass of injection (of which I am still uncertain as to the means by which it enters into the cellular structure) is washed out, the rami nutritii will become evident. The rami nutritii of the spongy substance (which, since they are upon the walls of the sinuous veins in the interior of the penis, may be also called the vasa vasorum) are found to be as minute as the arteries of any other part: they distribute themselves upon the pillars of the spongy substance, until they become too fine to be perceived by the naked eye. As in the arteries of other parts, they anastomose; and lastly, they form, as in other parts, the capillary net-work which is so difficult to be injected in the penis, owing to the facility with which the injection escapes into the cavities of the corpora cavernosa.

II. *Arteriæ Helicinæ Corporis cavernosi* (in Man.)

In order to see these arterial branches satisfactorily, an injection composed of size and vermillion must be thrown into a separated penis, through the art. profunda. (In the horse the pudendal and obturatorial artery are to be injected together.) As before mentioned, a part will escape into the cavities of the corpora cavernosa. When the injection has become cold, the corpora cavernosa must be cut open longitudinally, and that portion of the injection which has escaped into the cells is then to be washed out with great care. If a size of a greater degree of consistence has been employed, it will be found to have become solid on cooling. In this case the penis must be soaked in water, and the mass squeezed out softly and carefully, until the cellular tissue is emptied. When a thin size has been used, this will, of course, not occur; then washing alone will be sufficient.

If the tissue of the corpora cavernosa be now examined with a magnifying lens on its posterior third, it will be seen that, in addition to the distribution of the arteries already described, there is another class of vessels, having an entirely different form, size, and distribution; these branches are short, being about a line in length, and a fifth of a millimetre in diameter; they are given off from the larger branches, as well as from the finest twigs of the artery. Although fine, they are still easily to be recognized with the naked eye; they come off from the artery mostly at a right angle, and projecting into the cavities of the spongy substance, they either terminate abruptly, or else swell out into a club-like process, without again subdividing."

Omitting the references to these vessels in the stallion and the dog, we may proceed with M. Mueller's description of them in man.

"These twigs branch off from place to place, sometimes alone, sometimes in greater number: little bundles will be seen, in which from three to ten twigs stand together; these, as well as the former project constantly into the cells or venous cavities of the corp. cavern. penis. When the arteries thus form a tuft, they arise by a common stem, which immediately divides itself into the separate branches. Sometimes such a vessel, whether it proceeds from the artery as a single branch, or forms part of a cluster, divides itself into two or three parallel branches, which also end either abruptly, or else swell out near their extremity.

Almost all these arteries have this character, that they are bent like a horn, so that the end describes a half circle, or somewhat more. When such a branch so divides itself, there are formed doubly bent twigs, inclined one to the other. I have before observed, that many of these arteries enlarge towards their end; this enlargement is gradual, and is greatest at some little distance from the extremity, so that the end is somewhat conical. This cone, however, is rounded at the point, and, giving off no branches, terminates immediately. The diameter of these twigs, in their middle, is from the fifth to the sixth of a millimetre: they preserve a great similarity: thus those which branch off from the large trunk of the artery are not thicker than those which take their origin from the finest subdivisions."

Although these vessels project into the venous cavities, yet they are not entirely naked, but possess a delicate membranous covering.

"The arteries have no openings which can be detected, either on their surface, or at their extremities; and if the blood, as it is probable, proceeds from them during erection in greater quantity into the cells of the corp. cavernosa, so it must either pass through invisible openings, or at least through openings which only become enlarged by the great extension of the vessels. If the great number of these tendril-like branches which are given off from the art. profunda penis be considered in comparison with the many fine nutritive twigs of the same vessel, it must be evident that when the former are filled, they must take up by far the greater portion of the blood conveyed by it. The diameter of the art. profunda, therefore, not only includes the nutritive twigs which arise from it, but also the tendril-like branches, which likewise deriving their blood from it, yet, it is probable, allow none to pass except during erection; therefore the blood in the unexcited state of the penis only pervades the nutritive branches, and thus only reaches the commencement of the venous cells in smaller quantities; whereas, during erection, it is probable that the blood passes in quantity through these tendril-formed vessels into the cells."

M. Mueller states that these vessels are most numerous in the posterior part of the corpora cavernosa penis, and in the bulb. In the middle and anterior portions of the former, they occur but seldom, and in the anterior part of the corpus spongiosum urethræ they are less frequent; in the glans, M. Mueller has not yet distinguished them.

Such is the account of M. Mueller. These minute anatomical investigations can be prosecuted by so few, that the statements of a discoverer must be taken for granted, until some industrious injector or dissector arises to confirm or to confute them. As the phenomenon of erection is by no means confined to the posterior part of the corpus cavernosum, it appears rather anomalous that there only these vessels, so essential to that phenomenon in the opinion of our author, should be numerous. This difficulty may, perhaps, be more apparent than real, and we shall congratulate M. Mueller on his discovery, if it is supported by the examinations of others.

---

### Mr. Ure on the Chloride of Zinc, as a Remedy for Cancer.

Mr. Ure, formerly house-surgeon to the Glasgow Infirmary, and a very zealous and intelligent young gentleman, has lately been directing the attention of the profession to the use of the chloride of zinc, in cases of malignant ulceration.

Dr. Canquoin, of Paris, has published some papers on the best mode of preparing and applying this substance. Dr. C. would appear to have coquetted at first somewhat quackishly upon the subject, publishing his success before he gave any very accurate account of the means by which he attained it. His excuses are not so satisfactory as could be wished. But let this pass. Mr. Ure observes, in reference to the preparation of the remedy, that—

"Chemists offer the three following modes of preparing the chloride of zinc, which I have made the subject of comparative experiments:

1st, The neutral solution of zinc in muriatic acid may be evaporated to dryness, and fused. Or it may be obtained, 2dly, By distilling in a retort a mixture of one part of zinc with four of corrosive sublimate. Or, 3dly, by exposing to a strong heat, in an earthen retort, a mixture of six parts of decrepitated sea-salt with seven of dry sulphate of zinc; this process requires a red heat to separate the chloride by double decomposition. The product of distillation is of a greyish-white colour, translucent like wax; it enters into fusion a little above 212° Fahr., and becomes, on cooling, at first viscous, then solid."

Its chemical effects on the tissues of the body depend, in a great measure, on its power of coagulating and combining with albumen. Mr. Ure ascertained the following facts.

When a few drops of a strong solution of the muriate of zinc are added to the albumen of the egg, a white viscid compound is formed, which, when put into water, falls to the bottom, and remains undissolved. A layer of this substance closely resembles the eschar following the application of the phagedenic paste to an ulcerated surface; and it dries in the air into a brittle, horny mass.

The solution of muriate of zinc is a good test of albumen, though not so delicate as that of corrosive sublimate.

A single drop of it produces cloudiness in a dilute solution of gelatine.

With serum of blood it forms a viscid cream-coloured compound, analogous to that with white of egg. The liquid expressed from serum of blood coagulated by heat, becomes turbid on the addition of the muriatic solution.

A portion of the crassamentum, previously washed in distilled water, and then macerated in a solution of one part of the salt to ten of water, acquires in the course of some hours a firm coriaceous consistence and aspect, not unlike hepatized lung, or spleen that had been for a while immersed in the antiseptic saline solutions of the anatomist.

If the chloride of zinc, either by itself or in the form of paste, as proposed by Dr. Canquoin, be applied to an ulcerated surface or portion of the chorion, stripped of its epidermic covering, it constitutes with the organic texture, replete in albumino-gelatinous matter, a white inanimate crust or eschar. The great affinity of the chloride for albumen, may in some measure account for its energetic action on scirrhous and other morbid formations in which that principle predominates. From seven to eight hours are requisite for effecting this alteration. The slough is afterwards detached, like other sloughs, by an ulcerative process, the separation usually occurring in from eight to twelve days, or even sooner. The painful sensations occasioned by the application of the chloride of zinc usually cease in twenty-four hours. The eschar is white and very firm.

The four following formulæ are given by Dr. Canquoin, for the preparation of the paste.

I. Chloride of zinc one part, wheat-flour two parts.

II. Chloride of zinc one part, wheat-flour three parts.

III. Chloride of zinc one part, wheat-flour four parts.

IV. Chloride of zinc one part, chloride of antimony half a part, wheat-flour two parts and a half.

Twenty-four to thirty drops of water are to be added for each ounce of chloride.

The preparation of the phagedenic paste requires the utmost care and attention; hence, to procure it properly the following instructions must be scrupulously followed. The chloride of zinc, reduced to powder, is to be mixed, as quickly as possible, on a slab, with the given quantity of flour. One-half of the mixture is immediately to receive its proportion of water, and to be worked up progressively with a spatula, until it forms a homogeneous paste like honey. This paste is to be brought to the desired stiffness by trituration with the remainder of the dry ingredients, well beat for a few seconds, and then rolled out into cakes or wafers, of from half a line to four lines in thickness.

The quantity of water must be proportionally augmented, according to the increased amount of flour in the second and third formulæ.

The antimonial paste, No. IV., is to be moulded into a crayon shape; because, as it preserves constantly the consistence of soft wax, a suitable thickness can always be given to it, so as to adapt it to the form of certain cancerous tumors, presenting inequalities of surface.

Mr. Ure thought that it would be better to substitute some inorganic powder for the flour, the spontaneous chemical changes in which might possibly be prejudicial. He found that the anhydrous sulphate of lime, in a state of purity, answered very well. The paste so formed resembles putty, and is perfectly plastic.

The following is the manner of using the paste.

"Where the integuments are sound, the epidermis should be removed by means of a blister; and on the following day, one or other of the above preparations, corresponding to the thickness of tissue to be destroyed, is to be applied to the cutis of the diseased part. The sensibility of the surface must also be considered; for should it possess but a feeble degree of vitality, the most powerful form is to be preferred.

The paste No. I. four lines thick, applied during four days, is capable of producing an eschar of from one and a half to two inches in depth. The same paste, three lines thick, applied during three days, will furnish an eschar of one inch at least in depth; the same compound, two lines thick, will in two days determine an eschar of not less than half an inch. The paste No. I. of one line, will yield, in twenty-four hours, an eschar of three lines. Finally, the paste No. I., of half a line, will produce, in the same time, an eschar of at least one line.

These changes will manifest themselves with the above precision only on tissues endowed with a considerable share of sensibility, and of which the consistence is nearly normal. In the gristly (lardacé), almost fibro-cartilaginous degeneration, about one-third is to be deducted from the thickness of the eschar above-mentioned.

No. II. is employed in cases of cancerous ulceration and superficial carcinoma, which are attended with much pain.

No. III. is eligible in every species of cancerous affection, occurring in nervous subjects, who are incapable of supporting the violent pain which the preceding more concentrated escharotics might occasion. It is so much less productive of suffering, as it is slower in its action.

Lastly, the antimonial paste is best adapted to nodulated cancerous tumors, for which a more decided escharotic action is required.

These preparations, applied over a denuded surface, excite, in a few minutes, a feeling of heat, which, ere long, rises to a burning heat; which unpleasant symptoms may be relieved by an opiate enema.

When the operation of the paste is complete, it may be gently taken off, and the eschar covered with an emollient poultice until its separation, which usually happens, as formerly stated, from the eighth to the twelfth day, according to the thickness of the layer employed. The application is to be repeated again and again, till the whole morbid structure is removed; after which the surface is to be treated with simple digestive ointment; or, in case of acute cancer, with cataplasms, until the cure is finished."

The preceding are the principal circumstances necessary to be known by those who would test the value of the chloride of zinc. We would offer one or two remarks upon the subject.

We fancy that few practical surgeons in this country will believe, for one instant, that it is a remedy for cancer. An escharotic cannot be so sure a means as the knife of eradicating a morbid growth. Yet, even where amputation of a limb has been performed, how frequently do we witness a re-appearance of the disease in other parts. In the majority of cases of scirrhus or medullary sarcoma, if any operation at all is proper, that operation should be the removal of the affected part by the knife. It is more certain, more expeditious, and far less painful than the use of any caustic can be.

But there are some ulcerations possessed of a less degree of malignancy than scirrhus or medullary sarcoma. Such ulcers occur about the face of old persons, as well as in other situations. They are malignant enough to be irremediable by any measures short of those productive of destruction to the part; yet they do not contaminate the constitution, nor are other parts or other tissues affected, after the removal of the primary ulcer or growth.

The chloride of zinc promises to prove an exceedingly useful application in cases of this nature, and Mr. Ure deserves much credit for introducing it to the notice of the profession in this country so ably as he has done.

---

Some Cases of Disease of the Kidney, in which the Symptoms were referred to the Bladder. By Henry James Johnson.

Sir Benjamin Brodie, in a note appended to the first edition of his Lectures on the Diseases of the Urinary Organs, has made the following observations.

"On referring to the remarks which I have made under the head of *Irritable Bladder*, in the Fourth Lecture, I find that I have been guilty of a serious omission, inasmuch as I have not noticed the disordered sensations which are referred to the bladder, in many cases of disease of the kidney.

In speaking of renal calculi, I have mentioned the case of a lady, in whom a too frequent desire to void the urine, attended with a sharp cutting pain in the act of voiding it, was manifestly the consequence of a calculus impacted in the kidney; and many other cases have fallen under my observation, since my attention was called to the subject, which have convinced me that it is not uncommon for a patient, who labours under a stone, or abscess, or even inflammation of the kidney, to describe his symptoms as if they belonged to the bladder, which is free from disease, rather than to the organ in which the disease is actually situated. It is needless to point out how important a knowledge of this fact is to the practical surgeon. Those who are unacquainted with it, or whose minds are not constantly open to the probability of its occurrence, cannot fail to mistake the nature of a certain number of the cases concerning which they are consulted, to the no small detriment of their patients.

In these cases, in addition to the irritable state of the bladder, and pain referred to the neck of the bladder and urethra after micturition, there is usually a dull pain in the region of the affected kidney, extending downwards and forwards to the groin and pubes, or down the thigh. Occasionally there is pain referred to the corresponding testicle; and I have even known the testicle to be affected with a slight degree of inflammation, swollen, and sometimes indurated. The urine is generally acid, but slightly turbid even when first voided, and exhibiting a considerable quantity of albumen, when exposed to heat, or on the addition of nitric acid. In some cases the urine deposits a purulent sediment. On introducing a catheter, no obstruction is perceptible in the urethra, and the patient is found to be capable of emptying the bladder by his own efforts; and when the bladder is examined by means of a sound, no calculus can be detected in it. In the progress of the disease, it not unfrequently happens that there is evidence of an abscess of the kidney bursting into the ureter, there being a sudden discharge of pus and blood with the urine, preceded by symptoms similar to those which mark the descent of a calculus from the kidney into the bladder; and I have known masses of coagulated lymph, laminated, and of a cylindrical form, as if moulded to the shape of the ureter, to be discharged in the same manner.

It is by attention to the foregoing circumstances that we are enabled to distinguish between the irritable state of the bladder, which depends on disease of the kidney, and that which depends on other causes. Nevertheless, we are sometimes perplexed in our diagnosis, so that it is only after a careful investigation of the history and present symptoms, and after having for some time watched the changes which take place, that we are enabled to arrive at a satisfactory conclusion as to the real nature of the case."

The preceding hints well deserve the attention of practical surgeons. Cases of the nature described by Sir Benjamin Brodie are not very uncommon, and the symptoms are calculated to mislead an inexperienced or incautious person. As I have met with several cases of the kind, and had lately the care of a very interesting one, perhaps the details may not be unacceptable to the readers of this Journal.

*Case* 1. A boy, 16 years of age, was sent to me by a physician, for my opinion, in the early part of the year 1833. He was of rather a strumous appearance and habit, but was stout and seemed to be in excellent health.

He complained of frequent desire to make water, with great irritation in the course of the urethra. The act of micturition was attended with acute pain in that part of the urethra contained in the glans penis; the pain lasting for a short time after micturition was completed. Small quantities of urine were voided at a time, but the stream was never suddenly arrested. In consequence of the frequency of making water, and of the pulling and pressure to which the penis was subjected, in order to deaden the pain in the urethra, the organ had become much elongated. The urine was pale, and clear—acid—and slightly albuminous. There was no pain in the back, either with or without pressure, nor any retraction or uneasiness of the testicle.

On sounding the patient, I could detect no calculus in the bladder. Much pain was complained of, when the instrument came in contact with the trigone, and on making pressure on this part of the bladder from the rectum, the lad experienced considerable uneasiness. The prostate was of its natural dimensions. The bladder was capable of emptying itself perfectly.

The symptoms had commenced some months previously, and had gradually grown worse. The boy had been sounded several times, but no calculus had ever been discovered. There was no obvious cause for the disorder, which was considered by those who had attended him one of "irritable bladder."

The severity of the symptoms, in conjunction with the absence of calculus in, or appreciable disease of the bladder, and the albuminous condition of the urine, induced me to conclude that the case was one of affection of the kidney. What that affection was, there were no means of ascertaining. I wrote to the gentleman who had sent the boy to me, acquainting him with this opinion, and expressing an apprehension that the result would be fatal.

After a short time the boy was placed under my care. The symptoms had augmented in intensity, but were the same in kind. No more than a wine-glassful of urine could usually be retained in the bladder—the pain in voiding it was absolutely excruciating—and day and night the poor boy was roused to make water every hour or hour and a half. The quantity of albumen in the urine was increased. Still no alteration of the bladder could be detected by the most careful examination.

It would be useless to enumerate the various remedies that I employed.—None were productive even of much palliation to the sufferings. Viewing the case as most probably one of chronic inflammation of the kidney, I tried frequent small cuppings on the loins, and subsequently friction with the tartar-emetic ointment. This plan, with the exhibition of pills consisting of the milder narcotics, as lettuce and conium, and occasional warm-baths, seemed to give more relief than any other. Any thing in the shape of a diuretic medicine aggravated all the symptoms. The uva ursi, the dandelion, the buchu were injurious. Exhausted with the incessant calls to make water, and with pain, the boy's health and strength rapidly declined, and his friends at length removed him to his native county, Norfolk. They had an insuperable objection to the idea of an examination after death, and they did not let me know of the patient's decease until some time after its occurrence. Seven or eight months elapsed between the period of my first seeing the boy and his death.

I presume that this was really a case of disease of the kidney; and although dissection neither established its actual nature, nor indeed the correctness of the opinion at all, yet the similarity of the two succeeding cases in their most important features, will probably remove all doubt from the minds of the most sceptical.

*Case* 2. A gentleman, about 50 years of age, was under the care of Mr. Bagster and my Father, for just such symptoms as have been detailed in the preceding case. They were so like those of stone in the bladder, that Sir Benjamin Brodie was requested to sound the patient. He did so, but could discover no calculus.

Suddenly the gentleman was seized with symptoms of acute peritonitis, and in a day or two he died.

The body was examined in the presence of Sir Benjamin Brodie and Mr. Bagster. There were the usual results of violent peritoneal inflammation—lymph and puriform fluid. But both were tinged of a bilious colour, and it was found that the bile had escaped from the gall-bladder into the peritoneal cavity, through a small ulcerated opening in the former. This explained the peritonitis and the sudden death.

The urinary organs were carefully examined. The bladder was scarcely, if at all, altered. Perhaps its mucous membrane was rather darker than it should be, but no farther change had taken place.

The right kidney displayed no material alteration. The left kidney, however, was far from healthy. Its capsule could be detached with much more facility than natural. The substance of the kidney was very dark coloured, from the dilated and loaded condition of its blood-vessels, and its structure was softer and more lacerable than it should be. The pelvis of the ureter and several of the infundibula were dilated, and the mucous membrane lining them injected, and evidently in a state of chronic inflammation. In the substance of the kidney, a calculus of the mulberry kind was impacted; it was lodged in a sort of cyst. In another part of the kidney was a circumscribed abscess, about the size of a filbert, without any calculous deposition in it.

The preceding case is highly interesting on this account; accidental circumstances permitted an examination of the body in an early stage of the disease. When this has continued for any length of time, chronic inflammation is set up in the bladder—the disease in the kidney itself becomes both extensive and complicated—changes, perhaps, take place in contiguous or in distant parts—and, the exact nature of the primary affection is distinguished with difficulty, if it can be so at all, amidst its numerous and serious consequences.

In the next case the disease proceeded to its final termination, and its details may enable us to form an estimate of the effects it may give rise to.

*Case* 3. In August of last year, a gentleman, about 55 years of age, consulted my Father, on account of what he thought was an affection of the bladder. My Father referred the gentleman to me, in order that I might ascertain the state of that organ. The following were the symptoms and history of the case.

There was frequent desire to make water, the patient being obliged to void it every two or three hours during the day, and twice or thrice during the night. The quantity passed at a time was small, seldom exceeding half a pint, and generally not amounting to more than an ounce and a half or two ounces. The urine was straw-coloured, and not perfectly pellucid, though it could not be said to be turbid. Here and there was seen floating in it, a minute thread, apparently, of lymph. On testing it, the urine was acid, and decidedly albuminous.

There was much pain prior to, in, and after, but principally in the act of micturition, the pain being referred to the perineum, and the portion of urethra contained within the glans. The irritation experienced both in the bladder and urethra, unless the desire to make water was complied with, appeared to be excessive. After the urine was discharged, the patient felt ease for ten minutes or a quarter of an hour, at the end of which time, the irritation and pain usually recommenced.

The exit of the urine was never abruptly stopped, nor was any blood discharged with it. There was no pain in the loins, either with or without pressure; but occasionally a little uneasiness had been felt, in the lower and back part of the left side of the chest.

The general health was not very good, the digestive organs being easily deranged. The tongue was habitually furred, the bowels apt to be confined, and there was a disposition to a sense of confusion in the head. The countenance was rather pinched, sallow, and unhealthy.

On introducing a sound into the bladder, I could detect no calculus. There was some tenderness about the trigone and prostate, but no perceptible enlargement of the latter. The bladder was able to empty itself completely, or very nearly so.

This gentleman had been an employé in a government office, and had always, from his youth, been ailing. A few years before I saw him, he had been obliged to retire from his office, on account of bad health—I believe affection of the head. His present symptoms had commenced about nine months back, and had been progressively, though gradually, getting worse. Just before he came under my care, he had been to a very eminent surgeon in London, who sounded the bladder, and was inclined to think that serious disease of it existed.

From the general character of the symptoms, the albuminous urine, and my inability to detect a calculus in the bladder, or any alteration in its structure, I came at once to the conclusion that the case was one of disease of the kidney, and that the irritability of the bladder was merely symptomatic of renal affection. My Father arrived at the same opinion, and we communicated it to the friends, to whom we also hinted the probably fatal issue of the malady.

We directed the abstraction of small quantities of blood by cupping from the loins and perineum—mild aperients—warm water enemata, with hyoscimus and laudanum—occasional suppositories of lettuce and belladonna—passive exercise—and country air. For a short time the patient improved. But the amendment was transitory only, and the symptoms became slowly more and more severe. The uva ursi, with or without the liquor potassæ, and henbane, greatly increased the quantity of urine, and aggravated the distress. In October, I had the pleasure of meeting Sir Benjamin Brodie on the case. He took the same view of its nature, and recommended a steady persistance in the plan of treatment adopted. Unfortunately no material benefit was obtained, and about Christmas, I joined Dr. Prout in consultation. His opinion coincided with our's, and advised the establishment of a seton in one loin. This I made, but it produced such excessive irritation, and so alarmingly reduced the patient, that, after endeavouring to retain it for a fortnight, it was necessarily withdrawn.

The patient was now in a pitiable condition. He was obliged to make water every half hour during the day, voiding from half an ounce to an ounce at a time. At night he was obliged to rise for the same purpose from ten to fourteen times, or oftener. The irritation in the penis and the perineum was extreme—the urine was highly albuminous, and retained its former acidity. The patient was absolutely worn down with suffering—anxious—emaciated—feeble.

It would be useless to particularize the medicines that were employed.—Those which gave most ease were sedatives by the mouth and rectum—cupping to a small amount upon the perineum—and belladonna plaisters on the perineum or the loins. Thinking that possibly direct applications to the mucous membrane of the bladder, might diminish its irritability, though sympathetic only, I injected into it warm water containing conium or lettuce in solution. At first, the injection was attended with much relief, but after two or three times it became useless.

In the latter end of January, the patient had the advantage of Mr. Liston's advice. He prescribed the alcoholic extract of nux vomica in minute doses. The symptoms were relieved for a few days, but, soon afterwards, the patient was attacked with fever, vomiting, great depression, and aphthæ. When I saw him, after a short interval, he was emaciated to a great degree—had incessant vomiting—hectic fever—and the urine contained a large quantity of pus.

In the course of two or three weeks the vomiting had subsided, and the aphthous condition of the mouth and throat was relieved. The hectic connued unabated, and the debility was excessive. Still the patient was under the necessity of being propped up every half-hour, or oftener, in order to make water, for he was unable to use the urinal. The quantity of pus in the urine was enormous, and for two or three weeks preceding his death, bubbles of sulphuretted hydrogen gas were discharged from the urethra. Twice or three times in the last three weeks, there was a temporary retention of urine, for which I was obliged to use the catheter. I found that an instrument, of a size that had formerly been introduced with facility, could not be passed through the membranous part of the urethra, and that great pain was experienced when the instrument arrived there. The poor gentleman was in such a state of misery and exhaustion, that I made no examination by the rectum, but contented myself with relieving him by a small catheter.

About a fortnight before death, we accidentally discovered a tumor of some size in the right iliac and lumbar regions. It was apparently contained in the abdominal cavity, and both my Father and myself concluded that it was the right kidney, either greatly distended with pus, or the seat of some other alteration. The stupor of the patient, his sufferings, and his utter repugnance to be touched, or even spoken to, prevented us from meddling with him in any way, or from making any examination but by stealth. We were under the necessity of keeping him continually under the influence of opium.

On the 5th of May he died.

*Dissection.* I examined the body on the 7th, in the presence of Mr. Liston —Mr. Hunter, of the guards—and my friend, Mr. Henry Charles Johnson.

The chest was opened first, and the whole of the right side was found full of pus. There were all the characters of empyema—several quarts of purulent fluid in the pleural sac—the costal and pulmonary pleura thickly coated with false membrane—and the lung compressed to very small dimensions, and flattened against the mediastinum. In the apex of the lung were two or three calcareous depositions; but in other respects its substance was healthy. Of course the air had been pressed out of it, and it felt, in consequence, like wetted chamois leather. The left lung was united to the side of the chest by extensive and old adhesions. It was healthy, with the exception of the apex, where, as in the other lung, a few calcareous depositions existed.

There was slight hypertrophy of the left ventricle of the heart, which was otherwise free from alteration.

On opening the abdomen, and removing all the viscera with the exception of the right kidney, this was found to constitute the tumor that had been distinguished during life. But I was surprised at its appearing much smaller than that tumor had seemed to be. This was presently explained. The kidney itself was about twice its natural size. Its natural structure was entirely destroyed, and it was little else than a congeries of abscesses, from the size of a marble to that of a walnut, enclosed in a greatly thickened capsule. The matter contained in these numerous cavities was thick, and rather curdy. One of the abscesses, seated near the posterior surface of the kidney, had opened by an ulcerated aperture into the lumbar fossa. Here there was an immense abscess, the parietes of which were formed by the common integument behind, the greater part of the quadratus lumborum and contiguous muscles being destroyed—in front by the peritoneum and fascia—by the vertebral column internally, the psoas being almost absorbed—by the diaphragm above—and below by the ligament of Poupart. This collection of pus it was, which, with the kidney, formed the large tumor that had been felt during the patient's life. But this was not all, for an ulcerated aperture was found in the diaphragm, near the last rib, sufficiently large to permit the finger to be passed through it. Through this opening the matter had made its way into the right side of the chest, and occasioned the empyema. So that there might be said to be one immense abscess from the summit of the chest to the groin—perforating the diaphragm—only prevented from bursting posteriorly by the mere skin, the great substance of the muscles of the back being absorbed—bathing the vertebræ—and originating in one of many abscesses in the kidney, with which it communicated by a distinct fistulous aperture.

The walls of the ureter were greatly thickened, but its channel was unobstructed, and no calculus was any where discovered.

The left kidney was enlarged and vascular, but not apparently diseased. As it had to do much of the work of the right kidney, we may suppose that the vascularity and enlargement were the consequence of increased exertion.

The bladder was not at all thickened, nor was there any evidence of chronic inflammation of its mucous membrane. But on the fundus of the bladder, and particularly on its left side, were a few minute projections of the mucous membrane, of the size of millet-seeds, and looking like very small cutaneous warts.

Round the membranous part of the urethra, was a small collection of yellow pus, situated in the cellular tissue, between the prostate and the bulb, and totally unconnected with either. The canal of the urethra was not perceptibly encroached on by this surrounding suppuration.

There was no other alteration worth mentioning discovered. The head, however, was not examined.

It cannot be doubted that this interesting case was, in its origin, one of chronic inflammation of the kidney, terminating in the formation of circumscribed abscesses. The discharge of the contents of one of the latter into the lumbar fossa, and the subsequent increase of the abscess in the latter, and *its* entrance into the chest, are all sufficiently intelligible. An abscess of the kidney, opening thus, would be more likely than an ordinary collection of pus to give rise to such results, as some urine must almost necessarily be mingled with the matter.

Whether the minute excrescences of the mucous membrane of the bladder contributed in any way to aggravate the symptoms, is difficult to be determined. Probably they exerted little influence, as we find symptoms equally severe, in other instances of alteration of the kidney, unaccompanied with any such condition of the bladder.

The ingress of the matter into the chest must have occurred towards the conclusion of the patient's life, when he lay in a state of mixed stupor and exhaustion. It gave rise to no symptoms calculated to excite suspicion or attention—a circumstance not uncommon in the latter stages of debilitating maladies. I recollect the case of a patient at the hospital, who had empyema after scarlet fever. The operation of paracentesis thoracis was performed, and he lay for some time before his death in a state of much exhaustion and of hectic. After death we found that, without any symptoms whatever during life, suppuration had occurred in one hip-joint, and consecutive dislocation of the head of the femur, which was greatly absorbed, had actually taken place on the dorsum of the ilium.

The collection of matter around the membranous part of the urethra would seem to have occurred, also, in the two or three weeks preceding death. It will be recollected that, about that time, retention of urine occurred twice or thrice, and that a small catheter only would pass. Why it should occur it is not easy to say; but, when large collections of pus exist in any part of the body, other collections are apt to form in other parts. The continual straining to make water must, of course, have been a source of irritation to the urethra, and rendered it more liable than would otherwise be the case to inflammatory action.

This case is probably deserving of attention, because, as I had an opportunity of tracing it from an early period to its conclusion, we are enabled to form an estimate of the consequences that may ensue from what seem to be but slight beginnings. If the symptoms of what is popularly called "irritable bladder" are conjoined with an albuminous condition of the urine, the surgeon should be aware that he may have a very formidable malady to contend with. There is reason to believe that it is not merely one, but several organic alterations of the kidney, that give rise to this train of symptoms and consequences. Yet there is also reason to suppose that, in most instances, there is a chronic inflammatory condition of the organ, and a tendency to suppuration in it.

In the majority of cases, medicine would appear to exert little influence on the disorder. But, as we are only imperfectly acquainted with its pathology, and as the cases themselves have not been sufficiently watched or understood, to allow of any decisive conclusions being drawn, it would be improper to decide that the complaint is not at all under the control of treatment. Indeed, there are good grounds for supposing, that cases have done well under judicious management. In some patients, the progress of the disease is very slow. A man was in St. George's Hospital nearly five years ago, with symptoms of a very aggravated character. He left the house apparently dying. About two months ago he called on me, in order to be sounded. He was much in the same condition as when he was an inmate of the hospital. He was under the necessity of carrying a gum catheter about with him, and he passed it for himself very often during the day in the street-corners. The only perceptible alteration, was his appearing to be 20 or 30 years older than when I had previously seen him.

In the cases that have occurred under my own observation, the remedies that were productive of most relief were such as may be deemed adapted for chronic inflammation of the kidney. Frequent cuppings to a small amount on the loins or perineum—the application of a tartar-emetic plaster to the loins, or the establishment of an issue or a seton—the hip-bath—injections of warm water or of opiates into the rectum—mild aperients—regulated diet—and passive exercise. But it must be owned that rational as means of this description may appear, they are too commonly attended with only temporary benefit, or with none.

---

### Dr. Ryan's Formulary of Hospitals.

*To the Editor of the Med. Chir. Review.*

*A——h, May* 18*th*, 1836.

Sir,—Having occasion to refer to your very valuable Journal for October, 1835, I observed in the Bibliographical Record the announcement of "a new Practical Formulary of Hospitals, &c. translated from the French of Milne Edwards and P. Vavasseur, by Dr. Ryan," and to which you have appended high recommendation. In consequence of this I procured the book a week or two ago, not, however, without some misgivings, I must confess, when I saw the name of Henderson as the publisher. On examining this new Formulary of hospitals, my fears were realized; and I feel convinced that the character given of it in your Review must have been inadvertently inserted, without due examination of the book.

It is, without exception, one of the most inaccurate, and, consequently, useless publications ever presented to the medical public. If it were, however, merely useless, it might pass unnoticed; but, if the directions be followed, it must prove highly dangerous. The value of a work of this kind consists mainly in its accuracy; and the want of that is inexcusable, where the labour of the writer is only that of translation, or of mere transcription.

To shew the inaccuracy with which this work has been got up, I have cursorily looked it over, and of the many hundred formulæ which it contains, I believe that not less than one-sixth are manifestly erroneous. Probably many more are inaccurate, for I have only marked such errors as are palpable; *e. g.* where ounces are put for drachms, and vice versa—or where the weights are altogether omitted—or where the directions for the use of one article apply to another—or where the substances blended together are incompatible, or impossible to be mixed. To make these blunders appear still more ludicrous, a list of twenty-two errata is given, to shew that the work has been carefully revised—and even these errata contain errata!

To prove these assertions it will be necessary to go a little into detail; and, as there is scarcely a page in the book without an inaccuracy, I will make the selections at random—first from the translator's own formulæ, and then from his translated ones.

*Page* 262, we have the "anti-dysmenorrhœal mixture" (Ryan).

"℞. Misturæ camphoræ.. ℥vss.

Tinct. *Guiaci.* am. .. ℥vj.

Liq. ammon. acet. . ℥j.

Liq. opii sedat...... ʒj.—jss.

Syrupi aurantii .... ℥j.

Fiat mistura: de qua unum cochleare amplum mane vespereque exhibeatur.

This mixture is often beneficial in painful menstruation, caused by irritable uterus in hysterical women. A tea-spoonful of laudanum is an old remedy in this country. Guaiacum is strongly recommended by Professor Dewees, of Philadelphia, and acetate of ammonia by Professor Masuyer, of Strasburg, and M. Cloquet, of Paris. I have, therefore, combined them."

I have copied this note as a specimen of our author's method of prescribing. He finds one substance recommended by one writer—a second by another—and a third by some one else. He therefore sagaciously combines them all together, forming a quartum quid, which of course unites all their good qualities. I will only extract one more of Dr. Ryan's own choice formulæ.

"Aphrodisiac lozenges" (Ryan), *p.* 280.

"℞. Panacis, *v.* Fol. pulv. ℥v.

Vanillæ aromat..... ℥x.

Succini essentiæ .... ℈ss.

Tincturæ lyttæ...... ℥v.

Olei cannellæ ...... ♏l.

Sacchari purif...... ℔xij.

Mucilag. acaciæ. q. s. Fere intime et divide in pastillos, gr. xxiv., ex quibus sumantur tres vel quatuor ter quaterve de die."

This seems to be a favourite subject with our author; for, although he states that "it is doubtful whether there are any aphrodisiac remedies," he yet subjoins a somewhat indecent commentary of several pages on aphrodisiacs in general, drawing hints and analogies from the means used for increasing the generative powers of stallions and bulls—the methods pursued by the Jews for this purpose, &c.

Of the translated formulæ we have, in almost every page, such specimens as this.—*Page* 129.

"Anti-scrofulous potion—Hôt. Dieu.

℞. Tincturæ gentianæ.. ʒj.

Ammoniæ carbonatis ʒss.

Misce.

The carbonate of soda is sometimes substituted for the ammonia. *Externally*, as a stimulant and resolvant."

No directions are given for the internal use of this potent potion. On turning to Ratier's Formulaire, I find the potion anti-scrophuleuse of the Hôtel Dieu, contains ℥j. of the tinct. gentian to ʒss. of ammonia or soda. But he adds to this, as to all his other formulæ, a useful note :—" Plusieurs des médecins de l'Hôtel Dieu, persuadés que les scrophules s'accompagnent d'un état inflammatoire, que les toniques ne peuvent qu'aggraver, emploient un traitement tout opposé."

*Page* 380.—" Submuriate of bismuth: in doses of a drachm, an irritant poison—in small doses, a very efficacious sedative in neuralgia of the stomach and intestinal canal." He selects only two formulæ for this mineral.

" Sedative powder—Hôtel Dieu.
℞. Bismuth. subnitr... gr. iv.
Magnes. calcia....
Sacch. purif.... āā gr. xl.
In chartulas iv. divide. Capiat j. singulis horis."

This is a correct translation of the "poudre contre la gastrodynie"—Hôtel Dieu, M. Recamier; and, if this be the proper dose for Frenchmen, the next formula would seem to shew that the Americans require stronger medicines.

" Tonic pills—H. of America.
℞. Bismuthi subnitr.... ℥ij.
Mucilag. acac. q. s. fiant pilulæ xxxvi. Dosis j. secundis horis."

If one drachm of the subnitrate of bismuth be, as the author says, an irritant poison, half a drachm every two hours must be a hazardous remedy.

*Page* 251.—" Sulphate of potassa; an energetic stimulus. In large doses it is a violent poison—in small ones, it is a stimulant of all the organs, but more especially of the skin, the lungs, and the organs of circulation." A number of formulæ of this substance are given, *e. g.*

"Sulphuric Potion.—Hosp. of Germ.

℞. Potassæ sulphatis.... ʒj.

Aquæ.............. Oj.

Sacchari............ ʒij.

Fiat Poti, cujus capiat cochleare magnum. Recommended in poisoning by arsenic."

One spoonful of this solution must be as efficacious in poisoning by arsenic, as the following boluses are in mercurial salivation.

"Boluses used in Mercurial Salivation.—Hosp. of Italy.
℞. Potassæ sulphatis.. gr. iij.
Succi sambuci, q. s.—fiant Boli sex, quorum sumat unum tertia quaque hora."

How these potent boluses are to be made is left to the skill of the compounder. Several other similar formulæ are given, professing to be copied from the French hospitals: but it is the sulfure de potasse (sulphuret) and not the sulphate as it is here rendered.

Even the most common medicines are so travestied in this work as to become quite ridiculous. Of calomel, for instance, we have page after page of such formulæ as these.

*Page* 321.—"Anti-syphilitic opiate."
"℞. Hydrarg. submur... ʒij.
Opii purific........ xiv.
Conf. rosæ ........ ℥iv.
About the size of a bean of this opiate should be taken in the morning fasting, and a cupful of the decoction of guaiacum should be administered immediately after each dose, to prevent salivation."

On the same page we have "purgative pastiles."

"℞. Hydr. submur. .. gr. vj.
Chocolatæ ...... ℈j.
Fiat trochisci c. quibus capiat unum omni nocte.
Very useful to purge infants."

Whether the xiv. in the former prescription means drachms or grains, or whether the latter is to be made into one pastile, as the "fiat" would seem to imply, is left to the sagacity of the prescriber.

Dr. Ryan states in his preface, that "this work includes every medicine described in the British Pharmacopœias;" but I have in vain looked for many of the most efficient formulæ even in the London Pharmacopœia, and many of those which he has transcribed are inaccurate, vide liniment. hydrarg. &c.; and the calculation of the quantities of the efficient articles contained in them is equally so, vide Pil. hydr. subm. comp. &c. The index of the work, too, is so carelessly made as to be nearly useless. Even the errata, as I have above observed, contain errata. I need only instance one.

*Page* 55, first formula, *read* gutt. iij. Now, on referring to this page, there are no formulæ containing drops. On the next page we have this prescription:

"Collyrium of the *act*ate of lead—Hôpital de la Charité."

℞. Liquoris plumbi acet. gutt.

Aquæ puræ ........ ℥iv.

Ft. collyrium.

The resolvent collyrium of the Hôp. des Enf. differs only from the preceding, in the water being replaced by the infusion of elder flowers, and the acetate of lead being ʒj. to Oj. of liquid."

The above, then, is the prescription, I suppose, referred to; but, even in this simple formula, and the accompanying note, there are four errors.—1st, the reference is incorrect; 2dly and 3dly, the ingredient, and the quantity in the collyrium of the acetate of lead is wrong; the true formula of the Hôp. de la Charité being—

Aquæ (eau commune) ...... ℥iv.
Plumbi acet. (acet. de plomb.) gr. x.

and, 4thly, the resolvent collyrium of the Hôp. des Enfans is not a solution of the acetate of lead, but is as follows:

Infusion de fleurs de sureau.. ℔j.

Acetate de plomb. *liquide* (liq. plumb. acet.)............ ʒj.

Ft. collyrium.

It would be an ungracious and useless task to pursue farther these inaccuracies, which indeed present themself in almost every page; and one cannot but wonder what object the translator could have in giving to the medical public such a work. It mutilates and injures the character of the original; and is in itself worse than useless. After such models as Thompson's Conspectus and Ratier's Formulaire, it is trifling with the time and the pockets of medical men to obtrude on them such a publication as this. Some of Dr. Ryan's practical remarks are, it must be confessed, valuable. Some of his prescriptions are also unexceptionable; and if, instead of giving indiscriminately this farrago of useless and obsolete formulæ, he had made a careful selection from the foreign hospitals—from the prescriptions dispersed throughout your Journal, and other works of established character—from the London and provincial hospitals—as well as from private practice—he might have compiled a work of the highest practical utility, meriting the eulogium with which he has with great complacency characterized the present. "This Pocket Remembrancer," he says, "is in my opinion infinitely superior to, and more useful than, any other Conspectus of the Pharmacopœias or of prescriptions in our language."

I am, Sir, &c. &c.

T. P.

*To Dr. Johnson,*

*&c. &c.*

The above correspondent has given us his name and address. He has been rather too severe on the translator, and we have struck out several of his strictures. In justice to the public, we could not conscientiously refuse publication of this letter, as it will cause greater vigilance, and insure greater accuracy in a future edition. The transcription and correction of a volume of pharmaceutical formulæ, indeed, require such a sacrifice of time and labour, that we are astonished Dr. Ryan should embark in such a work as the above, considering the multiplicity of his avocations, professional and literary. Under all circumstances, Dr. Ryan should immediately examine the work, and print a full list of the errata, which purchasers can refer to and correct with their pens in the proper places.—*Eds.*

